# Antibacterial Creams Containing Cationic Carbosilane Dendrimers for Wound Treatment

**DOI:** 10.1021/acsapm.5c01718

**Published:** 2025-07-23

**Authors:** Rebeca Lozano-García, Sara Quintana-Sánchez, Selma Benito-Martínez, Guillermo Torrado, Víctor Guarnizo-Herrero, Borja Martínez-Alonso, Gemma Pascual, Bárbara Pérez-Köhler, Javier Sánchez-Nieves, F. Javier de la Mata

**Affiliations:** † Universidad de Alcalá (UAH), Departamento de Química Orgánica y Química Inorgánica, Edificio de Farmacia, Research Institute in Chemistry “Andrés M. del Río” (IQAR), Campus Universitario, 28805 Alcalá de Henares, Madrid, Spain; ‡ Networking Research Centre for Bioengineering, Biomaterials and Nanomedicine (CIBER-BBN), Instituto de Salud Carlos III, 28029 Madrid, Spain; § Institute Ramón y Cajal for Health Research, IRYCIS, 28034 Madrid, Spain; ∥ Universidad de Alcalá (UAH), Departamento de Medicina y Especialidades Médicas, Campus Universitario, 28805 Alcalá de Henares, Madrid, Spain; ⊥ Universidad de Alcalá (UAH), Departamento de Ciencias Biomédicas, Facultad de Farmacia, Campus Universitario, 28805 Alcalá de Henares, Madrid, Spain

**Keywords:** antibacterial, quaternary ammonium, carbosilane dendrimer, topical cream, skin wound

## Abstract

Skin wounds are an important factor in developing bacterial infection, especially for chronic wounds. In this case, the exposure to long traditional antibacterial-based treatments can lead to the appearance of resistance to these drugs. This situation makes the search for alternatives to attack these infections essential, as it is the use of cationic multivalent systems. Here, we discussed the antibacterial and biological properties of different cationic carbosilane (CBS) dendrimers against () and () as models of Gram-positive and Gram-negative bacteria, respectively. Dendrimers are a type of multivalent molecule with a well-defined structure. The CBS dendrimers used in this work differ in several modifications that affect the hydrophobic/hydrophilic balance, which is very relevant to achieve bactericidal activity. These structural changes are the position of a short alkyl chain, in the internal dendritic structure or on the outer ammonium groups, the presence of a polyethylene glycol (PEG) chain instead of a cationic function, or in the vicinal moieties of the cationic functions, sulfur atoms or sulfone units. The studies allowed the selection of some dendrimers, all of them with the inner long chain and trimethylammonium (−NMe_3_^+^) groups, as active ingredients of a topical cream (water in oil, W/O). The antibacterial and biological properties of the creams were also tested against bacteria because it is the most common pathogen involved in skin infections. We observed different abilities of the dendrimers to be released from the cream, depending on the dendrimer structure, and as a consequence, different antibacterial properties of the creams. Finally, an analysis of the physicochemical properties of the best formulation was also done.

## Graphical abstract

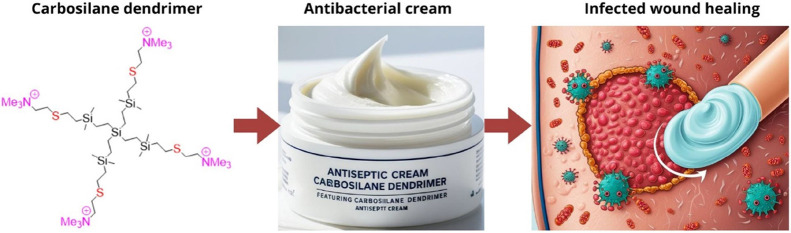

## 1 Introduction

The largest organ of the human body is the skin, which acts as a protective barrier from the external environment.^1^ This protective layer is the most exposed to chemical and physical impacts, which may provide a simple or severe disruption to this organ. Wounds activate the healing process to regenerate a functional epidermis and its underlying dermis and subcutaneous tissue.^2,3^ An improper repair process can lead to skin or soft tissue infection (SSTI).^4^ SSTI is one of the most typical infections that require medical intervention and contribute to morbidity and mortality in both primary care and hospitalized patients.^1,5^

Despite recent advances in wound treatment, microbial infections are still a major health issue. This fact is due to the capacity of microorganisms to develop resistance against microbicide agents, mainly antibiotics. Pathogenic microbes in wounds can proliferate and begin colonization, leading to biofilm formation.^6^ Bacteria and fungi in biofilms create an extracellular matrix, increasing resistance and hindering wound healing.^7^ Hence, improving the prevention and treatment of minor infections would allow reducing the overuse of antibiotics, which favors the origin of resistance and decreases the number of hospitalized patients with severe SSTI.^8^ In this way, topical antiseptic agents are advocated as a promising alternative to control infections at the outermost surface of the skin and reduce the unnecessary use of antibiotics. Only for skin and wound infections in the deeper skin layer, antibiotics should be prescribed.^4^

Antiseptics are characterized by their broad-spectrum activity against different microbes; however, at the same time, they are nontoxic and do not damage healthy tissue.^9^ Additionally, they do not exert any specific inhibitory mechanism, and consequently, bacteria cannot develop resistance easily. For this reason, topical antiseptics bring the possibility to prevent infections in wounds, killing bacteria or minimizing the growth of diseases caused by bacteria in the skin.^10^

Some of the chemical molecules used as antiseptic creams for the treatment of SSTI are silver compounds, quaternary ammonium compounds (QAS), octenidine dihydrochloride, or chlorhexidine digluconate. However, they present some limitations related to toxicity in healthy tissues or even the emerging risk of developing novel resistances following an extensive use of antimicrobials, and it is necessary to conduct research on new topical formulations with other active ingredients or modifications to reduce toxicity.^11^

In response to this demand, dendrimers have emerged as potential polymeric macromolecules with biocidal activity. The well-defined shape and structure, as well as the monodispersity of these macromolecules, give them good advantages over traditional polymers.^12,13^ Due to their multivalence, they can be conjugated with antimicrobial groups (QAS^14,15^ and others^16–18^ ), increasing their biocide effect in contrast to the activity of these individual molecules and modifying their pharmacokinetics due to their nanosize. Moreover, these systems can be heterofunctionalized with molecules of different nature, giving them multifunctionality and thus new properties for their subsequent application, e.g., ammonium and peptide,^19^ and ammonium and silver nanoparticles.^20^

Different types of dendrimers with antimicrobial activity have been described in the literature, depending on the core, polyamide (PAMAM),^21^ polypropylenimine (PPI),^22^ polyester,^23^ or cationic amphiphilic dendrons.^15,24^ The framework of these dendrimers is hydrophobic and contains hydrophilic fragments to facilitate their antibacterial activity. Our research group has studied the properties of carbosilane (CBS) dendritic systems, whose framework is highly hydrophobic.^25^ The adequate hydrophobic–hydrophilic balance is achieved by adding external QAS groups. The hydrophobic CBS skeleton favors the dendrimer penetration into the cell membrane, whereas the charge allows the displacement of the divalent cations that compose this membrane.^26–28^ In that way, it is important to note that the most active CBS compounds are usually of low generation.^29,30^ The greater dendritic generation, the greater steric hindrance, and consequently, the poorer ability to interact with the cell membrane.^28,31^ Moreover, the nonspecific mechanism of action of cationic dendritic systems prevents the development of bacterial resistance.^30,32,33^ The importance of an appropriate hydrophobic/hydrophilic balance for the antibacterial activity is supported by the behavior observed in other types of active dendrimers and polymers.^14,15^

Among the most active CBS dendrimers, highlight derivatives with a thioether group near the ammonium functions, but only with the adequate length of the internal alkyl chain of the dendrimer, since derivatives with a very short inner alkyl chain were not active as antibacterials due to the lack of the hydrophobic skeleton.^14,34^ On the other hand, the sulfur atom of the structure can be involved in biological processes, such as oxidation by oxygenases, forming toxic sulfoxides.^35,36^ However, this oxidation ability can also be beneficial for the cells since the thioether moiety in the amino acid Met is fundamental for the cells to remove reactive oxygen species.^37^ Regarding the ammonium group, an important drawback is the toxicity associated with this group. To minimize this problem, strategies as the introduction of polyethylene glycol chains or masking the cationic charge have been employed.^32,38^

With the aim to find a dendritic system with enhanced antibacterial properties and minimal toxicity drawbacks, to surpass the problems of antibiotic treatment in skin wounds, in this work, we have evaluated different modifications in CBS cationic dendrimers. One of them is the modification of the hydrophobic/hydrophilic balance with the introduction of longer alkyl chains in the inner dendritic structure or on the outer ammonium group. This last change also covers the cationic charge, which could be helpful to minimize toxicity. Another transformation is the oxidation of the thioether units close to the ammonium groups to sulfone units to avoid the possible effect of oxidation of this last atom. The biocide capacity of dendrimers was evaluated against () and (), Gram-positive and Gram-negative bacteria, respectively. Also, the redox properties of dendrimers were compared, and toxicity assays were carried out in erythrocytes and fibroblast cells. After these experiments, selected dendrimers were included in the formulation of topical antiseptic water-in-oil creams (W/O), and their biological properties were tested, focusing on the bactericidal activity of the creams.

## 2 Results and Discussion

### 2.1 Synthesis of Dendritic Systems

A library of cationic CBS dendritic systems functionalized with ammonium groups has been employed for the studies carried out herein. Several modifications in the dendritic structure have been considered, such as the alkyl chain length on the ammonium group −NMe_2_R^+^ (R = Me, Pr, (CH_2_)_2_OMe) or in the internal CBS skeleton, the introduction of one biocompatible function (PEG units) instead of one cationic group,^34^ and finally, the presence of sulfone moieties instead of thiother ones.

The synthesis of dendrimers functionalized with different ammonium groups and a short inner alkyl chain derived from commercial tetravinylsilane was carried out following a simple general procedure, which facilitates access to the library of compounds used here (Scheme 1). Briefly, this strategy consists in the alkylation of the neutral amine dendrimer G_0_Si­(NMe_2_)_4_,^29^ with the corresponding alkyl halides RX (RX = MeI, CH_3_CH_2_CH_2_I, CH_3_OCH_2_CH_2_Br) and Cl^–^ anion exchange with an anionic resin (Scheme 1). All of these new compounds **1a**–**c** were obtained in good yield. ^1^H and ^13^C-NMR spectroscopy confirmed the transformations proposed (Figures S1–S3). The most relevant change was the shifting to a higher frequency of methyl groups bound to the N atom (from ca. 2.1 to ca. 3.2 ppm, ^1^H spectra). The remaining resonances were as expected according to the type of groups present in the dendrimer (see Section 4 and SI).

**Scheme 1:**
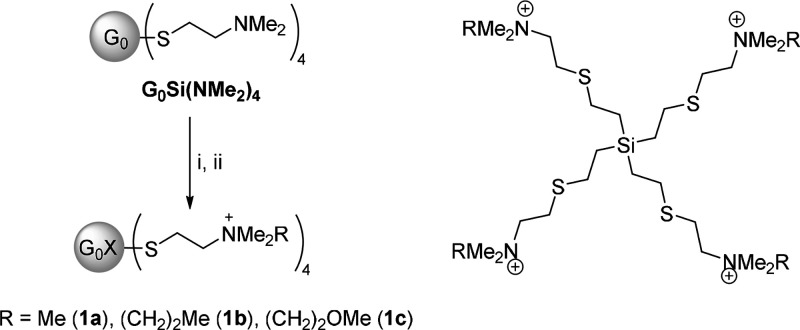
Synthesis of Cationic CBS Dendrimers with Different Alkyl Ammonium Groups and Drawing of the Structure of Dendrimers **1a**–**c** ^a^

a (i) For **1a**, 16 h, MeI, THF, r. T.; for **1b** Me­(CH_2_)_2_I, CH_3_CN, 60 °C, overnight; and for **1c**, MeO­(CH_2_)_2_Br, CH_3_CN, 60 °C. (ii) Amberlite-Cl, r. T., H_2_O, 2 days.

The other family of cationic CBS dendrimers contains a long inner alkyl chain and trimethylammonium groups (−NMe_3_^+^) (Figure 1). Compound **2a** ^34^ displayed four of these groups and a thioether vicinal moiety; similar to derivative **1a**, dendrimer **2b** changes one cationic charge by a biocompatible PEG2k unit,^34^ while dendrimer **2e** incorporates sulfone units instead of thioether ones. The PEG length is important for the biocompatibility of these dendrimers. We have chosen dendrimer **2b** for this work because we have observed that a related cationic dendrimer to **2b**, but with a shorter PEG chain (ca. 800 Da), exhibited similar antibacterial activity, although with much greater toxicity.^34^

**Figure 1:**
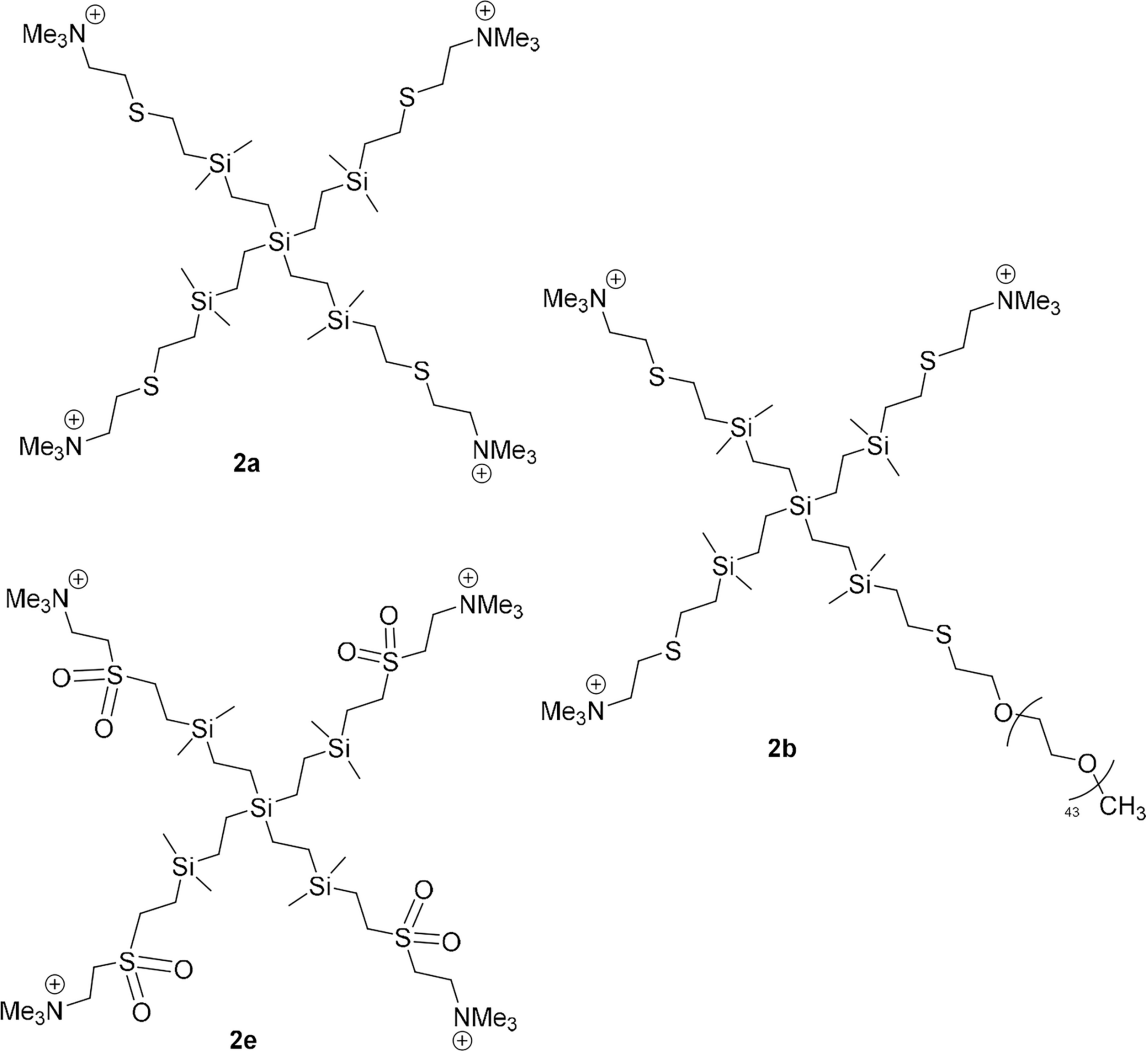
Drawing of the structure of cationic CBS dendrimers with a longer internal alkyl chain **2** **a**–**e**.

Compound **2e** was prepared (Scheme 2) following several steps. First, by oxidation of the thioether derivative with neutral amine functions, G_0_Si­(SiSNMe_2_)_4_,^34^ with oxone (potassium peroxymonosulfate, KHSO_5_·0.5KHSO_4_·0.5K_2_SO_4_), to form the corresponding neutral sulfone dendrimer (**2c**). Next, methylation of the amine groups with MeOTf forms the cationic dendrimer, and finally, triflate-chloride exchange allows obtaining the goal cationic CBS dendrimer (**2e**), with sulfone units and chloride counterions. The oxidation of thioether to sulfone moieties was clearly detected by ^1^H and ^13^C-NMR due to the shifting to higher ppm of the methylene groups bound to sulfur and nitrogen atoms (e.g., ^1^H spectrum, from 2.39–2.54 in G_0_Si­(SiSNMe_2_)_4_ to 2.78, 3.01, and 3.07 in **2c** (Figures S4 and S5). ^1^H and ^13^C-NMR spectra of **2e** again showed the presence of the new −NMe_3_^+^, shifted to higher ppm with respect to the starting neutral −NMe_2_ resonances (Figures S6 and S7).

**Scheme 2:**
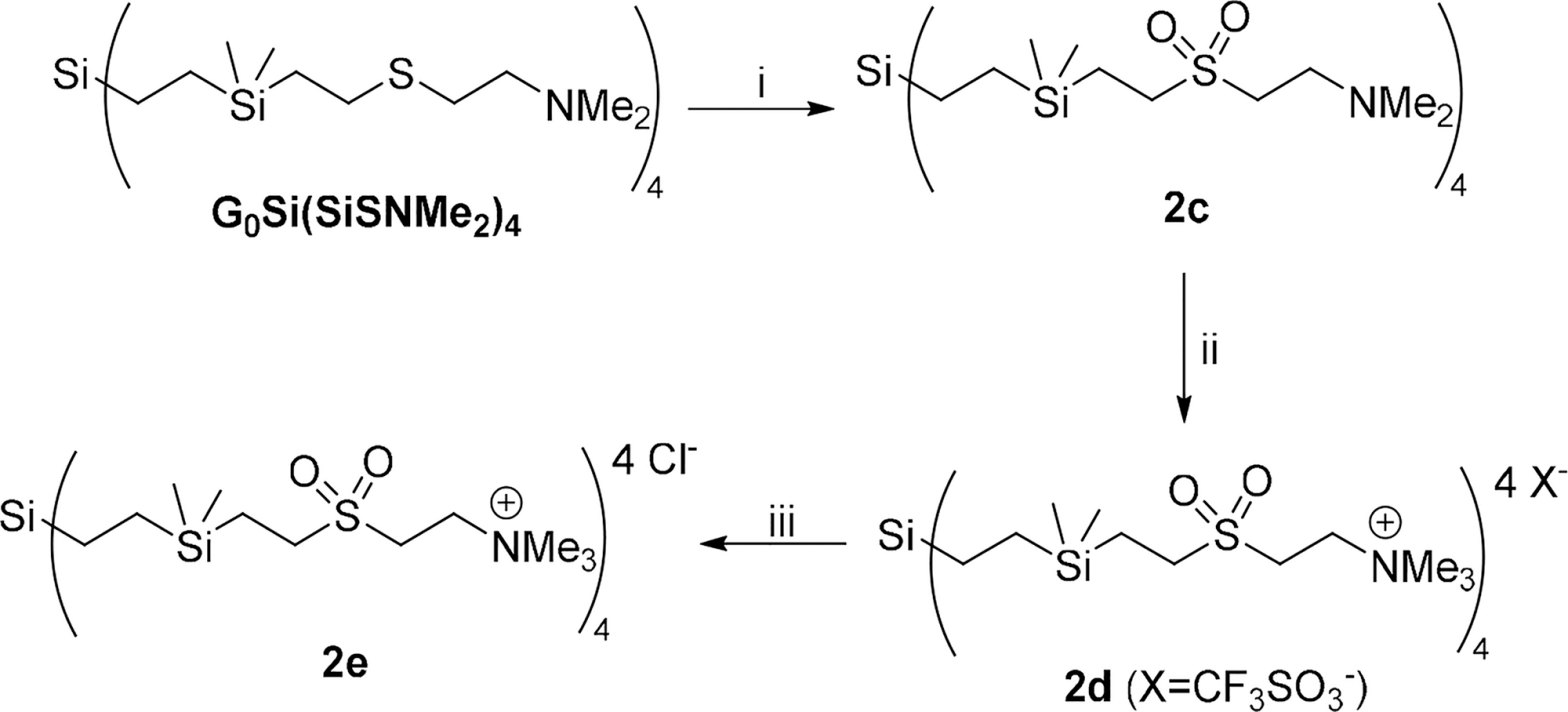
Synthesis of the Cationic CBS Dendrimer with Sulfone Moieties **2e** ^a^

a (i) Oxone (KHSO_5_·0.5KHSO_4_·0.5K_2_SO_4_), 0° C, 1 h; (ii) MeOTf, 24 h; and (iii) amberlite-Cl, r. T., H_2_O, 2 days.

The stoichiometry of the oxidation process was carefully adjusted. A lower proportion of oxone showed the formation of a mixture of compounds, probably corresponding to the formation of sulfoxide branches, but always dogged with sulfone units. If an excess of oxone was added, decomposition was observed. Also, the oxidation of the sulfur atom was explored with H_2_O_2_ and other peroxides (m-CPBA (meta-chloroperoxybenzoic acid), tBuOOH) under different conditions, observing the initial formation of mixtures of sulfoxide and sulfone branches, but then leading to decomposition without the possibility to isolate any sulfoxide or sulfone dendrimer. Regarding the transformation from neutral amine **2c** to cationic ammonium dendrimer **2e**, it is done by a typical methylation procedure; however, for this case, we had to use MeOTf instead of MeI since this last reagent led to decomposition of the dendrimer. This decomposition was also observed if the oxidation of the sulfur atoms was tried with cationic dendrimer **2a** instead of neutral G_0_Si­(SiSNMe_2_)_4_.

### 2.2 Antibacterial Activity of Cationic CBS Dendrimers

The antibacterial activity of dendrimers **1a**–**c** and **2a, b,** and **2e** was studied in (CECT 425) and (CECT 434) strains as models of Gram-positive and Gram-negative bacteria, respectively (Table 1).

**Table 1:** Antibacterial Activity of CBS Dendrimers **1a**–**c** and **2a**–**e** ^a^

					*MRSA*	
dendrimers	MIC	MBC	MIC	MBC	MIC	MBC
**1a**	256	512	128	512		
**1b**	256	256	256	512		
**1c**	256	256	256	512		
**2a**	4	4	1–2	2–4	2	2–4
**2b**	32	32	4–8	8–16	8	16
**2e**	128	128	8	16	8–16	16–32

a Minimum inhibitory concentration (MIC) and minimum bactericidal concentration (MBC) are given in ppm (mg/L). *MRSA*: methicillin-resistant .

These assays showed that modifications in the alkyl chain length and consequently in the hydrophobic–hydrophilic balance caused a remarkable increase in the bactericidal behavior of cationic CBS dendrimers, if the elongation of this chain is done in the internal framework structure (**1a** vs **2a**). However, if the elongation of the chain is done on the nitrogen atom of the ammonium groups, the bactericide activity is rather low and similar for all types of cationic groups, including −NMe_2_R^+^ (**1a**–**c**). This behavior is opposite to that observed for PAMAM^39^ and PPI^14^ dendrimers, whose bactericidal activity was higher for ammonium groups supporting long alkyl chains. These types of dendrimers contain highly hydrophilic cores that have to be equilibrated with these alkyl chains. For the subsequent studies, we discarded the very low active compounds **1a**–**c**.

On the other hand, the change of sulfur atoms by sulfone units decreased the antibacterial activity, highlighting the reduction in the strain (**2a** vs **2e**). Also, the substitution of one cationic charge by the PEG chain reduced the antibacterial activity (**2a** vs **2b**). This reduction in the activity can be associated with the significant reduction in the number of charges of this small dendrimer (reduction of 25%). Finally, dendrimers **2a**–**e** were tested against methicillin-resistant (*MRSA*, CECT 5190), showing similar values to those observed against nonresistant .

### 2.3 Redox Activity of Cationic CBS Dendrimers

As commented above, the presence of the sulfur atom in the dendrimer structure can be involved in oxidation processes, altering the redox conditions of bacteria and cells.^35,36^ In fact, the synthetic procedure of CBS with sulfone units **2e** is performed by oxidation of the sulfur atoms of a neutral precursor. For this, we have evaluated the redox properties of these three cationic dendrimers **2a**, **2b,** and **2e** in vitro. It is also important to note that usually the formation of the cationic charge (−NMe_3_^+^) requires the addition of MeI to the amine ligands, leading to the presence of iodide anions as counterions, which are later substituted by chloride ones. Thus, we have also evaluated the effect of these two anions in the redox behavior of **2a** (**2a-Cl** and **2a-I**) and **2b** (**2b-Cl** and **2b-I**). The assays carried out were DPPH-free radical-scavenging activity, which corresponds with single-electron and hydrogen atom transference, and ferric-reducing antioxidant power (FRAP), which corresponds with single-electron transference.

The results (Figure S8) indicated that the cationic dendrimers **2a**, **2b,** and **2e** lack redox properties regarding proton or electron transference, DPPH or FRAP method, respectively, since activity was only observed when the anion iodide was present (**2-I**), but not with the anion chloride (**2-Cl**). This data was confirmed by independent analysis of corresponding salts NaX (X = Cl, I) and is in agreement with the reduction potential of the anions. Hence, to avoid the influence of the counteranion on the biological studies, the cationic dendrimers studied are neutralized with Cl^–^ anions.

### 2.4 Biocompatibility Assays of Cationic CBS Dendrimers

To select dendritic systems of this library of compounds to prepare a microbicide cream, preliminary biocompatibility studies, hemolysis and cytotoxicity (fibroblast cells), were carried out.

According to the data in Table 2, the substitution of one ammonium charge by the PEG2k chain increases viability in RBCs (H50, **2a** vs **2b**), while the change of the sulfur atoms by sulfone units barely affected hemolysis (**2a** vs **2e**). This agrees with the higher toxicity of ammonium groups.^40^ However, considering these values and MBC (H­(MBC)), dendrimer **2e**, with sulfone units, was notably more toxic at active concentrations. Additionally, the selectivity index (SI) for **2e** calculated from data was clearly below 1, and close to 1 for , while for the other two dendrimers, they were above 1 for both bacterial strains. At MBC, dendrimers **2a** and **2b** did not produce hemagglutination; again, this last dendrimer with PEG units was clearly safer than the fully ammonium derivative **2a**. However, the sulfone dendrimer **2e** produced hemagglutination below its MBC for­(Figure S9), its value being similar to that of **2a**.

**Table 2:** Data of Biocompatibility for Dendrimers **2a**–**e** in Red Blood Cells (Hemolysis)^a^

dendrimer	2a	2b	2e
MBC	4	16	16
MBC	4	32	128
H50 (ppm)	28.3	96.6	28.0
H(MBC) (%)	8.21	8.24	31.4
H(MBC) (%)	9.92	18.2	100
hemagglutination (ppm)	32	256	64
SI	28.3	24.2	3.50
SI	7.08	3.02	0.22

a H50: concentration for 50% of hemolysis (ppm); H­(MBC): percentage of hemolysis at MBC; SI: selectivity index (SI = H50/MIC).

The toxicity of these dendrimers **2a**–**e** in fibroblast cells was analyzed at the highest MBC of both strains () for each dendrimer (Figures 2 and 3). From these assays, it is inferred that fibroblast cells were tolerant to dendrimers with sulfur units **2a** and with a PEG unit **2b** at their respective MBC, with 100% of viability. However, the dendrimer **2e** with sulfone units was very toxic, even at its lower MBC of .

**Figure 2:**
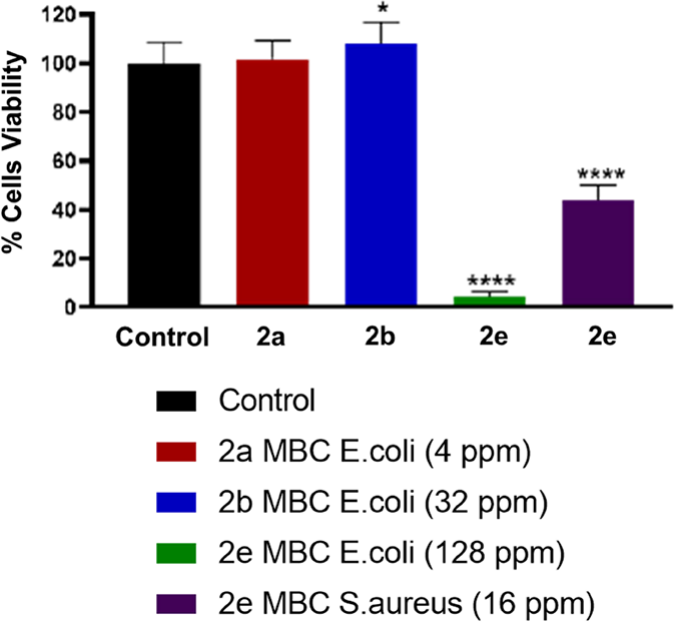
Determination of the percentage cell viability by means of the alamarBlue method. Graph represents the mean ± SEM of the different groups. Legends: * **2b** vs control (*p* < 0.05); **** **2e** vs control (*p* < 0.0001).

**Figure 3:**
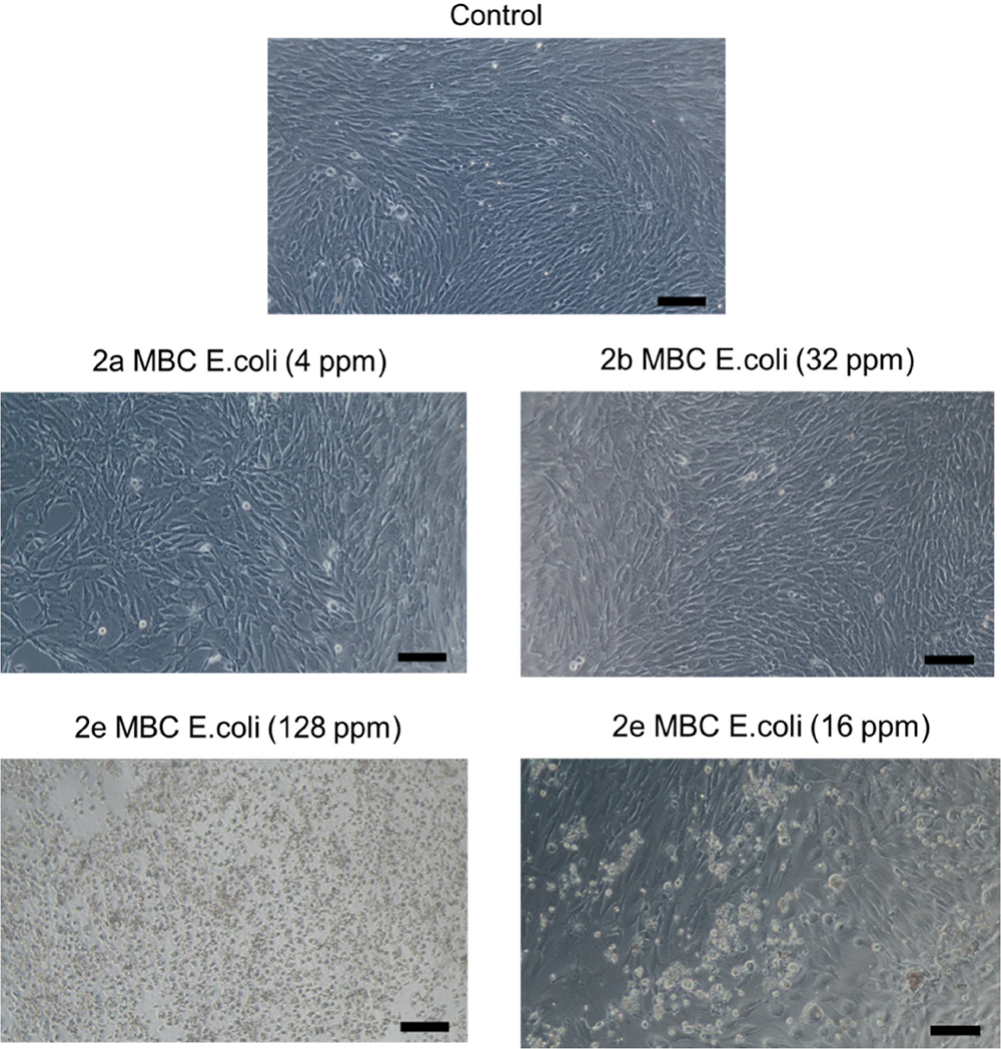
Light microscopy micrographs (100x, scales 100 μm) of cultured fibroblasts following a 24 h incubation under exposure to the CBS dendrimers **2a**–**e** at concentration equivalent to MBC recorded for (all) and (**2e**).

### 2.5 Formulation of Creams and Characterization

The three cationic CBS dendrimers **2a, 2b,** and **2e** were used as active ingredients of water-in-oil (W/O) creams at a concentration of 1%. The other ingredients of the cream were selected by their lack of bactericidal activity (bee wax 15%, SPAN 80 8%, mineral oil 47%, water 29%; see Section 4 for the synthetic procedure). According to the data obtained for the antibacterial activity of these creams (*vide infra*), the only active cream was cream **2a**. For this reason, this cream was the only one whose physiochemical properties were analyzed.

The organoleptic properties, including color, odor, physical appearance, and immediate skin feel of the cream placebo and cream **2a**, are displayed in Table S1. Results showed that the creams had acceptable properties, the only change in the color of the cream being the presence of dendrimer **2a**. This change has no effect on the cream's acceptability.

Spreadability refers to the ability of a semisolid formulation to cover a surface over time. This property is critical for ensuring that a consistent dose of the formulation is applied to the skin and for the overall effectiveness of topical treatments. Increased spreadability facilitates easier application.^41^ The spreadability tests performed on the creams in this study demonstrated favorable properties for both formulations (Figure S10); however, the presence of dendrimer **2a** as an active ingredient affected the cream’s behavior. This effect was more clearly observed through the spreadability factor, with the placebo cream exhibiting the highest spreadability factor among the formulations (Table S3). In any case, the extensibility values achieved are considered ideal for ensuring effective application of the formulation containing active **2a**.

Rheology provides quantitative information on the viscoelasticity of an emulsion. We herein explored the creams (placebo and **2a**) to evaluate the impact of the dendrimer in the formulation. Two main parameters were examined: the storage modulus (*G*′), which quantifies the material’s elasticity, and the loss modulus (*G*″), which measures its viscous behavior and ability to dissipate energy as heat. If *G*′ > *G*″, then the material can be considered mainly elastic. If *G*″ > *G*′, the material can be considered primarily elastic.

The measurements must be performed within the linear viscoelastic region (LVR), where the material can be deformed without losing its microstructural properties. We calculated the LVR through an amplitude sweep assay at 35 °C, keeping the frequency at 1.6 Hz and varying the oscillatory strain (OS) from 0.05% to 500%. A sudden decrease in *G*′ that eventually intersects with *G*″ indicates the critical strain point, where the material microstructure is altered and therefore loses its viscoelastic properties. As depicted in Figure 4A,B, the presence of the dendrimer in the cream did not modify the viscoelastic properties of the cream since the creams placebo and **2a** showed very similar critical deformations, both around 1%. Accordingly, we selected 0.1% OS as the optimum.

**Figure 4:**
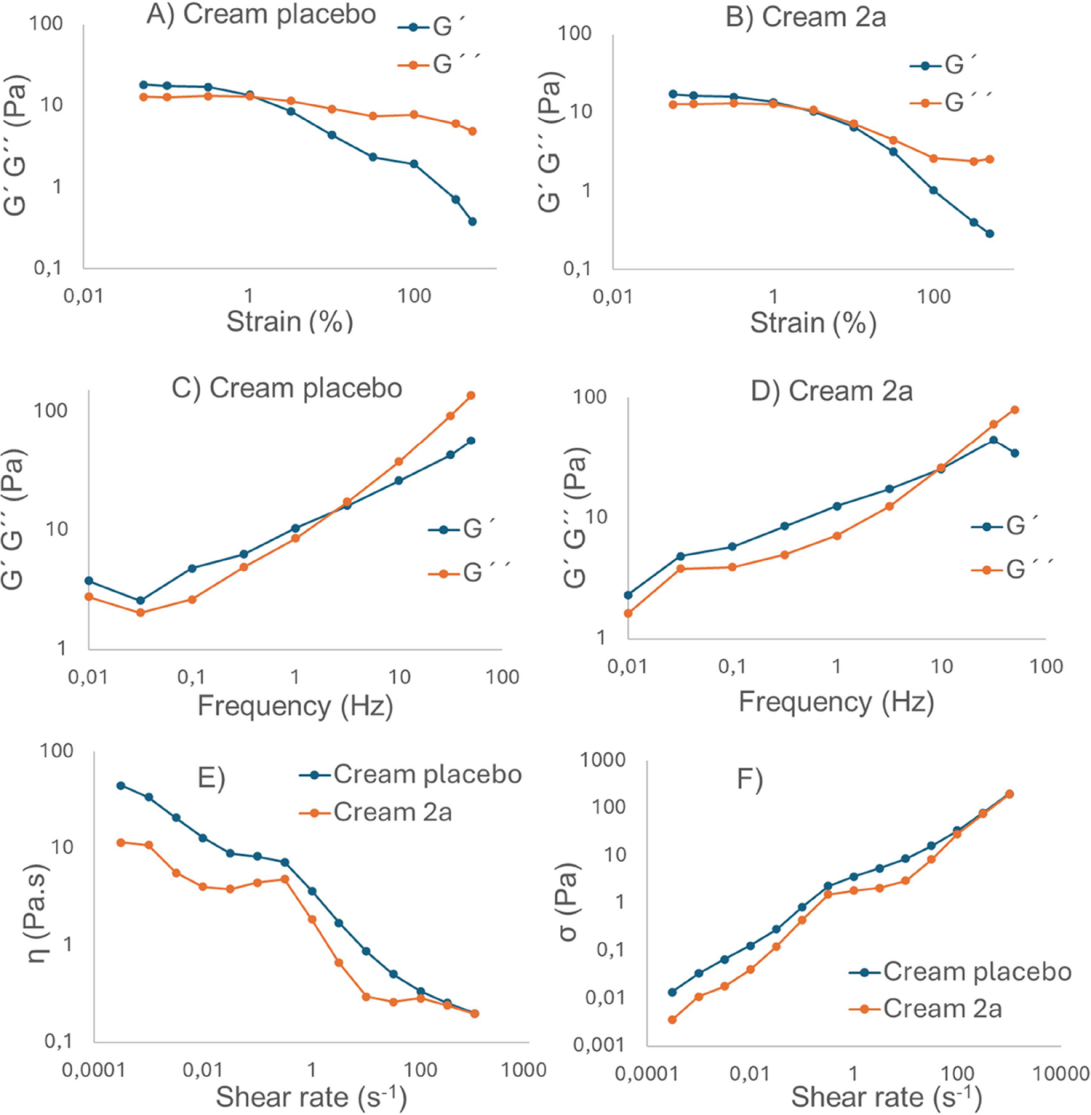
Rheological characterization. Storage (*G*′) and loss (*G*″) moduli in amplitude sweeps at 35 °C, with 1.6 Hz for the placebo cream (A) and cream **2a** (B). Storage (*G*′) and loss (*G*″) moduli in frequency sweeps at 35 °C with oscillatory strain 0.1%, for cream placebo (C) and cream **2a** (D). Flow characterization of creams placebo and **2a**: (E) viscosity, η; (F) shear stress, σ.

Once the LVR was established, the creams were studied through a frequency sweep assay. The assay was performed in the range of 0.01–50 Hz with the OS at 0.1% at 35 °C. This assay provides more information about the behavior of the materials (solid, liquid, or gel). As depicted in Figure 4C,D, both creams showed frequency dependency of the moduli, with dominant elastic behavior at low frequency and viscous behavior dominant at higher frequencies. The type and concentration of excipients will influence the rheological properties of the final product.^42^ Both *G*′ and *G*″ were similar in both creams, although the frequency of the crossover between *G*′ and *G*″ increases with the presence of the dendrimer, from 3 Hz for the cream placebo to 10 Hz for cream **2a**. Therefore, the crossover of *G*′ and *G*″ can be used as a critical process parameter for the final formulation.

Continuous shear experiments evaluated the ability of each system to withstand structural degradation under shear stress. Viscosity is defined as the material’s resistance to flow or deformation, which is influenced by shear rate, as well as the physicochemical properties of the formulation and temperature. Depending on their rheological characteristics, formulations can be classified as Newtonian or non-Newtonian.^42,43^ A Newtonian formulation maintains a constant viscosity regardless of shear rate, while non-Newtonian systems exhibit viscosity changes with varying shear rates. Non-Newtonian formulations can be pseudoplastic (shear-thinning), where viscosity decreases at high shear rates, or dilatant (shear-thickening), where viscosity increases under high shear conditions. Figure 4E,F presents the shear rate dependences of the apparent viscosity and shear stress for each emulsion. A decrease in apparent viscosity was observed with an increase in shear rate in the range of 10^–4^–1000 s^–1^, indicating that the formulations exhibit non-Newtonian behavior, specifically pseudoplastic. The pseudoplastic behavior of antiseptic formulations is advantageous for skin application, as it requires relatively low force to spread the formulation, which would minimize additional damage to the skin.^44^ This rheological behavior is characteristic of water-in-oil emulsions. In addition, the presence of the dendrimer in the cream reduced the viscosity of the cream at each shear rate.

### 2.6 Effectiveness of Topical Biocide Cream

The in vitro antibacterial activity of each cream was evaluated against and *MRSA* bacteria since these microorganisms are among the main pathogens responsible for developing wound infections.^45^

First, we tested the growth inhibition of both and *MRSA* in a liquid medium (Figures 5 and S11). For these, the creams were introduced in a six-well microplate in contact with a 10^6^ CFU/mL bacterial suspension. After 24 h at 37 °C, the final absorbance of the sample (white column in Figures 5 and S11) was compared with the initial one, observing an important reduction of the bacterial viability only for cream **2a**, that is, with dendrimer containing four ammonium −NMe_3_^+^ and sulfur vicinal atoms. For the other two creams, we rather observed activity. Similar behavior was observed for both strains (Figures 5 and S11). Longer exposure times of the suspensions to the creams did not affect the result. The antibacterial activity against of cream **2a** was repeated after 6 months of storage at 8 °C. This activity decayed by half after this period (Figures 5 and S11).

**Figure 5:**
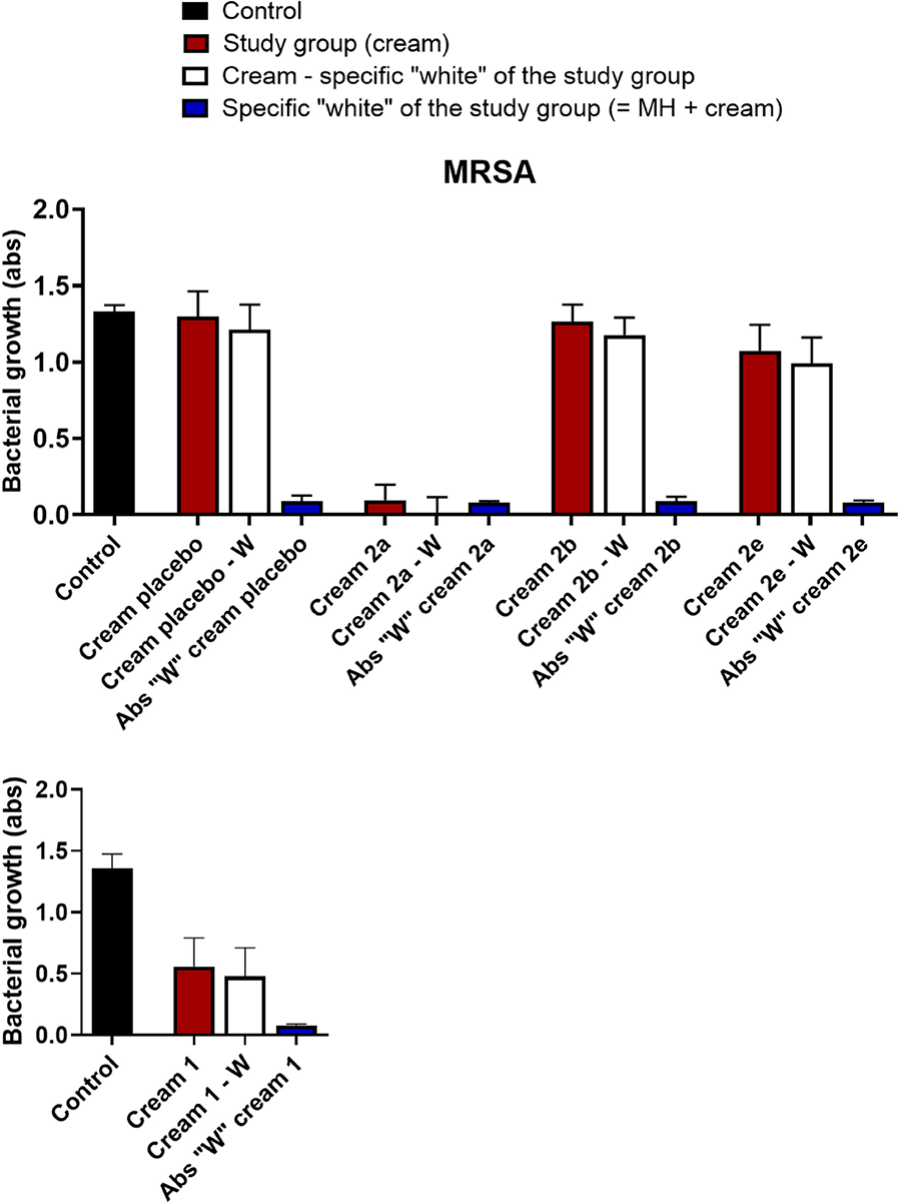
Bacterial growth of bacteria (*MRSA*) in MH broth after incubation with the creams **2a, 2b,** and **2e** at 37 °C for 24 h (top) and 6 months (bottom, only cream **2a**). In black, the absorbance of bacteria culture without treatment (positive control); in red, study group (cream), absorbance of bacteria culture after treatment with cream; in white, absorbance of only bacteria culture after treatment with cream (real activity, red minus blue); and in blue, absorbance of cream (negative control).

Second, we evaluated the area of inhibition, defined as the region without bacterial growth around a well punched in an agar plate, where the cream was deposited (Figures 6 and S12). This experiment corroborated the previous one, and only cream **2a** showed an inhibition zone after 24 h (in both strains). The data did not change after 48 and 72 h for the three formulations (Figure S12).

**Figure 6:**
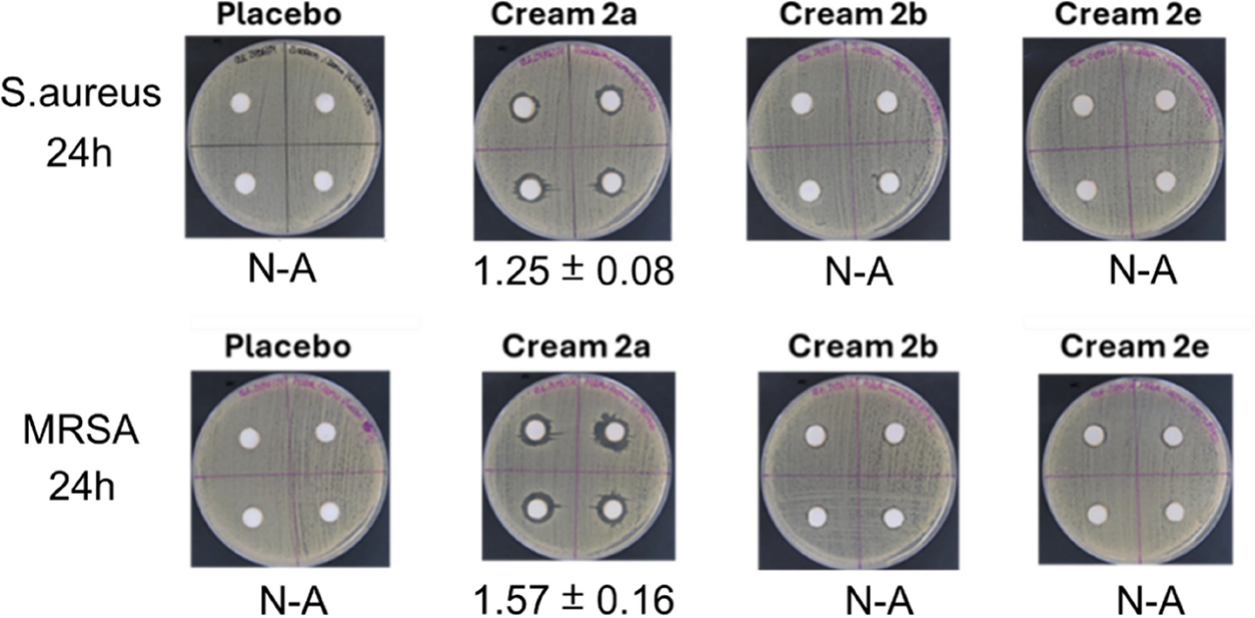
Agar-diffusion assays with topical biocide creams **2a**, **2b,** and **2e** in and *MRSA* (after 24 h). N-A: Non-antibacterial. Area of inhibition is in cm^2^.

A third check of the cream’s behavior was done to determine whether the dendrimer is released from the creams. For this, creams (100 μL, ca. 100 mg) were incubated with a saline solution for 24 h, and an aliquot of the supernatant was placed inside wells made in an agar plate. The diffusion halo was measured after another 24 h of incubation, confirming that only the solution from cream **2a** had antibacterial properties. If this procedure was repeated for the same cream sample but treated for 48 and 72 h, no halo was observed for any of the three creams. That is, cream **2a** released all of the possible dendrimer after 24 h (Figure S13).

This experiment was also employed to analyze the presence of the dendrimers by UV (see the Supporting Information). Of the three dendrimers, only dendrimer **2a** is detectable in the UV spectrum, with a maximum absorbance at 226 nm. However, this was not enough to observe the presence of dendrimer **2a** in the active solution since the MBC value is below the concentration threshold necessary to find **2a** in the UV spectrum (Figures S14 and S15). Therefore, dendrimer **2a** is released from the cream at rather low concentrations; however, due to its low MBC value, this concentration is enough to kill bacteria. For the other two dendrimers, we can consider that if dendrimers are released, their concentration is below the MBC.

A transdermal diffusion assay (see the Supporting Information) with Franz cells was done with Strat-M membranes for the cationic CBS dendrimer **2a** solved in a saline solution (ca. 60.25 ppm, which corresponds with the amount of dendrimer present in the release experiments described above). After 24 h at 37 °C, this experiment did not show modification of the concentration in the donor chamber, nor the appearance of dendrimer **2a** in the receptor chamber (by UV spectroscopy, Figure S16). It is very relevant that the lack of detection of dendrimer **2a** in this experiment means that, if any dendrimer trespasses this barrier, it will be clearly below the hemolysis H50 value since this concentration (28.3 ppm) is detectable by UV spectroscopy.

Finally, based on these results, we selected cream **2a** for the skin irritation test (OECD-439). The test system used in this study was the RhE model (normal human-derived epidermal keratinocytes). This analysis demonstrated that cream **2a** was nonirritant, with a viability of 100.5% ± 1.51 (Figure S17 and Table S4).

## 3 Conclusions

The elongation with short alkyl chains in the CBS dendrimer structure of small cationic dendrimers with four branches produces a positive antibacterial behavior if this elongation is introduced in the internal core (dendrimers **2a, 2b,** and **2e**) but not on the external ammonium groups. Additionally, the most active compound contains thioether moieties close to the ammonium functions. If one ammonium is replaced by a PEG chain or the thioether units are modified to sulfones, the antibacterial activity decreases. While the PEG chain favors the biocompatibility of the dendrimer, the sulfone units notably impaired the biocompatibility (hemolysis, hemoagglutination, and fibroblast viability).

The antibacterial properties of formulations containing dendrimers **2a, 2b,** and **2e** as active ingredients showed that only the cream with dendrimer **2a** retained bactericidal activity (dendrimer at 1%). Furthermore, this cream was not an irritant in an epidermal keratinocyte model.

Thus, we have demonstrated that the incorporation of highly active antibacterial but nontoxic (in terms of hemolysis and fibroblast cell viability) dendrimers into a cream can preserve its antibacterial activity without causing irritation. These findings could serve as a foundation for further studies on the potential applications of such systems as active ingredients in topical microbicidal creams, considering factors such as the dendrimer concentration, cream type, and additional biocompatibility assessments.

## 4 Experimental Section

### 4.1 Synthesis of Cationic Carbosilane Dendrimers

Compounds G_0_Si­(NMe_2_)_4_,^30^ **1a**,^30^ **2a**,^34^ and **2b** ^34^ were prepared as previously described. All reactions have been carried out under an inert atmosphere, and solvents were purified from appropriate drying agents when it was necessary. Commercial reagents were used as received.

#### 4.1.1 G_0_Si­(SCH_2_CH_2_NMe_2_PrCl)_4_ (**1b**)

Excess 1-iodopropane (0.80 mL, 8.1 mmol) was added to an acetonitrile solution of G_0_Si­(NMe_2_)_4_ (0.750 g, 1.3 mmol). The reaction mixture was stirred at 60 °C for 16 h. Afterward, volatiles were removed under vacuum. To exchange I^–^ anions to Cl^–^, the product was redissolved in distilled water and stirred in the presence of Amberlite IRA-402-Cl form for 16 h. Then, the solution was filtered to remove Amberlite, and water was evaporated by a rotary evaporator. Compound **1b** was obtained as a yellow solid (0.697 g). Data for **1b**: ^1^H NMR (CD_3_OD): δ = 1.07–1.16 (m, 20H, NCH_2_CH_2_C*H*_3_, SiC*H*_2_CH_2_S), 1.86 (m, 8H, NCH_2_C*H*_2_CH_3_), 2.83 (m, 8H, SiCH_2_C*H*_2_S), 3.04 (m, H, SC*H*_*2*_CH_2_N), 3.19 (s, 24H, N*Me*_2_), 3.39 (m, 8H, NC*H*_2_CH_2_CH_3_), 3.65 (m, 8H, SCH_2_C*H*_2_N). ^13^C­{^1^H}-NMR (CD_3_OD): δ = 11.81 (NCH_2_CH_2_ *C*H_3_), 14.92 (Si*C*H_2_CH_2_S), 18.02 (NCH_2_ *C*H_2_CH_3_), 25.94 (SiCH_2_ *C*H_2_S), 29.34 (S*C*H_2_CH_2_N), 52.01 (N*Me*_2_), 65.92 (N*C*H_2_CH_2_CH_3_), 67.72 (SCH_2_ *C*H_2_N). C_36_H_84_Cl_4_N_4_S_4_Si (871.20 g/mol): Calc.: C, 34.9; H, 6.8; N, 4.5; S, 10.3; Obt.: C, 34.6; H, 6.9; N, 4.9; S, 10.3.

#### 4.1.2 G_0_Si­(SCH_2_CH_2_NMe_2_((CH_2_)_2_OMe)­Cl)_4_ (**1c**)

Compound **1c** was prepared following the same method as **1b**. In this case, an acetonitrile solution of G_0_Si­(NMe_2_)_4_ (0.740 g, 1.3 mmol) was mixed with excess 1-bromo-2-methoxyethane (0.80 mL, 7.97 mmol). Afterward, Br^–^ anions were exchanged with Amberlite IRA-402-Cl form to obtain compound **1c** as an orange solid (0.766 g). Data for **1c**: ^1^H NMR (CD_3_OD): δ = 1.15 (m, 8H, SiC*H*_*2*_CH_2_S), 2.82 (m, 8H, SiCH_2_C*H*_*2*_S), 3.05 (m, 8H, SC*H*_2_CH_2_N), 3.25 (s, 24H, N*Me*_2_), 3.44 (s, 12H, NCH_2_CH_2_OC*H*_3_), 3.71 (m, 16H, SCH_2_C*H*_2_N, NC*H*_2_CH_2_OCH_3_), 3.87 (m, 8H, NCH_2_C*H*_2_OCH_3_). ^13^C­{^1^H}­NMR: δ = 12.54 (Si*C*H_2_CH_2_S), 24.13 (S*C*H_2_CH_2_N), 26.63 (SiCH_2_ *C*H_2_S), 50.43 (N*Me*_2_), 57.63 (NCH_2_CH_2_O*C*H_3_), 63.46–63.58 (SCH_2_ *C*H_2_N, N*C*H_2_CH_2_OCH_3_), 66.40 (NCH_2_ *C*H_2_OCH_3_). C_36_H_84_Cl_4_N_4_O_4_S_4_Si (1113.00 g/mol): Calc: C, 38.8; H, 7.55; N, 5.0; S, 11.5; Obt.: C, 37.1; H, 7.6; N, 5.2; S, 10.3.

#### 4.1.3 G_0_Si­(SiSO_2_NMe_2_)_4_ (**2c**)

Compound G_0_Si­(SiSNMe_2_)_4_ (0.889 g, 0.986 mmol, 1 equiv) was dissolved in CH_3_CN/H_2_O (1:2) and mixed with oxone (potassium peroxymonosulfate, KHSO_5_·0.5KHSO_4_·0.5K_2_SO_4_) (2.43 g, 7.89 mmol, 8 equiv) in an ice bath at 0 °C for 1 h. After 1 h, the reaction mixture was extracted three times. The aqueous phase with the protonated dendrimer and oxone was extracted and neutralized with an excess of Na_2_CO_3_. Then, we added CH_3_CN again, and the mixture was extracted in triplicate. The organic phase was extracted with a neutralized dendrimer and dried over Na_2_SO_4_. Compound **2c** was purified after solvent removal under vacuum conditions (1.003 g). Data for **2c**: ^1^H NMR (CDCl_3_): δ = 0.04 (s, 24 H, SiMe_2_), 0.40 (m,16 H, SiCH_2_CH_2_Si, SiCH_2_CH_2_Si), 1.04 (m, 8 H, SiCH_2_CH_2_SO_2_), 2.26 (s, 24 H, NMe_2_), 2.78 (m, 8 H, SiCH_2_CH_2_SO_2_), 3.01 (m, 8 H, SO_2_CH_2_CH_2_NMe_2_), 3.09 (m, 8 H, SO_2_CH_2_CH_2_NMe_2_). ^13^C­{^1^H}-NMR: δ = −3.99 (Si*Me*_2_), 2.65 (Si*C*H_2_CH_2_Si), 6.76 (Si*C*H_2_CH_2_SO_2_), 7.40 (SiCH_2_ *C*H_2_Si), 45.42 (N*Me*_2_), 49.69 (SiCH_2_ *C*H_2_SO_2_), 50.73 (SO_2_ *C*H_2_CH_2_NMe_2_), 52.67 (SO_2_CH_2_ *C*H_2_NMe_2_). C_40_H_96_N_4_O_8_S_4_Si_5_ (1029.89 g/mol). Calc.: C, 46.65; H, 9.40; N, 5.44; S, 12.35; Obt.: C, 48.54; H, 9.27; N, 5.24; S, 10.19.

#### 4.1.4 G_0_Si­(SiSO_2_NMe_3_Cl)_4_ (**2e**)

Compound **2c** (0.536 g, 0.52 mmol, 1 equiv) was dissolved in dry CH_2_Cl_2_ and mixed with methyl triflate (MeOTf) (0.683 g, 4.16 mmol, 8 equiv) in an ice bath at 0 °C for 24 h. Next, the solvent was removed under a vacuum to isolate **2d**. The product **2d** was solved again, without further treatment, in distilled water, and the CF_3_SO_3_^–^ anion was replaced by the Cl^–^ anion with Amberlite IRA-402-Cl form. Compound **2e** was obtained as a yellow solid (0.640 g). Data for **2e**: ^1^H NMR (CD_3_OD): δ = 0.10 (s, 24 H, SiMe_2_), 0.53 (m,16 H, SiCH_2_CH_2_Si, SiCH_2_CH_2_Si), 1.09 (m, 8 H, SiCH_2_CH_2_SO_2_), 3.18 (m, 8 H, SiCH_2_CH_2_SO_2_), 3.23 (s, 24 H, NMe3), 3.79 (m, 8 H, SO_2_CH_2_CH_2_NMe_3_), 3.86 (m, 8 H, SO_2_CH_2_CH_2_NMe_2_). ^13^C­{H} NMR: δ = −3.99 (Si*Me*_2_), 3.52 (Si*C*H_2_CH_2_Si), 6.66 (Si*C*H_2_CH_2_SO_2_), 7.97 (SiCH_2_ *C*H_2_Si), 46.44 (SiCH_2_ *C*H_2_SO_2_), 51.53 (SO_2_CH_2_ *C*H_2_NMe_2_), 54.09 (N*Me*_2_), 59.91 (SO_2_ *C*H_2_CH_2_NMe_2_). C_44_H_108_Cl_4_N_4_O_8_S_4_Si_5_ (1231.83). Calc.: C, 42.90; H, 8.84; N, 4.55; S, 10.41; Obt.: C, 39.81; H, 8.07; N, 3.99; S, 8.95.

#### 4.1.5 Nuclear Magnetic Resonance (NMR) Spectroscopy

All synthesized dendrimers were motorized and characterized by ^1^H, ^13^C­{^1^H} NMR, and {^1^H–^13^C}-HSQC-2D-NMR. NMR spectra were recorded on Varian Mercury Plus 300 and Bruker Avance III HD 400 instruments. Chemical shifts are given in ppm and measured relative to internal deuterated solvent peaks.

#### 4.1.6 Elemental Analysis

A LECO CHNS-932 instrument was used for elemental analyses.

### 4.2 Antioxidant Capacity

#### 4.2.1 DPPH-Free Radical-Scavenging Activity (DPPH = 2,2-Diphenyl-1-picrylhydrazyl)

In this assay, the DPPH radical is reduced by the antioxidant species from a purple color to a yellow color or a colorless species; therefore, it implies a reduction of the absorbance, with a maximum absorption at 530 nm.

For sample measurements, 180 μL aliquots of a previously prepared DPPH methanolic solution (111.11 μM) were placed in a 96-well plate. Subsequently, 20 μL of the dendrimers was added in methanol at concentrations of 10 to 100 μM, and the plate was kept in the dark at room temperature for 30 min. After that period, absorbance was recorded at 530 nm using a microplate reader (EpochTM, BioTek Instruments, Winooski, VT, USA). Methanol was used as a control. The scavenging activity was determined from the remaining DPPH for each dendrimer concentration, using the following equation:

$$[\mathrm{DPPH}{]}_{\mathrm{REM}}[\%]=100\times A_{s}/A_{c}$$

where REM = remnant, *A*_s_ = sample absorbance, and *A*_c_ = control absorbance.

#### 4.2.2 Ferric Reducing Antioxidant Power (FRAP) Assay

The FRAP method is based on the reduction at an acidic pH of the TPTZ (2,4,6-tripyridyl-striazine) (Fe^3+^-TPTZ) complex to the ferrous (Fe^2+^) form, developing an intense blue color with a maximum absorption at 593 nm. The ability of CBS dendrimers to reduce Fe^3+^ to Fe^2+^ was evaluated.

For sample measurements, 180 μL aliquots of a previously prepared FRAP stock solution in acetate buffer were placed in a 96-well plate. Subsequently, 20 μL of the dendrimers was added in methanol at concentrations of 10 to 100 μM, and the plate was kept in the dark at room temperature for 30 min. After that period, absorbance was recorded at 593 nm using a microplate reader (EpochTM, BioTek Instruments, Winooski, VT, USA). Methanol was used as a control. Preparation of the FRAP stock: Three solutions of TPTZ (10 mM solution in 40 mM of hydrochloric acid), FeCl_3_ (20 mM solution in ACS water), and acetate buffer (20 mM in 100 mL of ACS water, pH 3.6) were mixed in a 1:1:10 ratio. The Fe^2+^ formed was determined for each dendrimer concentration, using the following equation:

$$[\mathrm{Fe}(\mathrm{II}){]}_{\mathrm{FOR}}[\%]=100\times (A_{s}-A_{c})/A_{c}$$

where FOR = formed, *A*_s_ = sample absorbance, and *A*_c_ = control absorbance.

### 4.3 Antibacterial Activity

#### 4.3.1 Bacterial Strains

The antimicrobial activity validation assays were carried out with three bacterial strains ( (CECT 425) and *methicillin-resistant* (*MRSA*, CECT 5190) as Gram-positive strains and (CECT 434) as Gram-negative strain) obtained from the Spanish Type Culture Collection (CECT).

#### 4.3.2 MIC and MBC Assays

The antibacterial activity of several cationic dendritic systems against planktonic and has been evaluated following the international standard method ISO 20776-1:2006. Bacterial suspensions were diluted to a concentration of 2 × 10^7^ CFU/mL, while cationic dendrimer solutions were prepared in the range of 0.25–1024 mg/L in distilled water. Subsequently, treatments were carried out in triplicate in a 96-well microplate. After that, in each well, 100 μL of different concentrations of each biocide was mixed with 100 μL of double-concentration Muller–Hilton broth and 5 μL of bacteria inoculum. Positive control (inoculum in medium MH, without dendrimer) and negative controls (medium MH containing dendrimer and medium MH only) were added in all experiments. Samples were incubated for 20 h at 37 °C. Then, the minimal inhibitory concentration (MIC) was analyzed by measuring the absorbance at a wavelength of 625 nm at time 0 and 20 h of treatment in an ultramicroplate reader ELX808iu (BioTek Instruments). Being MIC considered for no turbidity wells, to determine the minimal bactericidal concentration (MBC), 5 μL of each treatment was incubated in an agar-PCA Petri plate for 24 h at 37 °C. The MBC was determined as the minimal concentration at which no bacterial growth was detected.

### 4.4 Biocompatibility Studies

#### 4.4.1 Hemolysis Assay

The hemolysis activity method, ISO10993-4 adapted, was used to determine the CBS dendrimer concentrations required to cause 50% hemolysis (H50) and H­(MBC) corresponding to the percentage of hemolysis at the MBC concentration.

The total red blood cells (RBCs) from 2 mL of defibrinated sheep blood were isolated by centrifugation at 800 g for 10 min and washed three times with PBS (phosphate-buffered saline, pH = 7.4). Then, RBCs were resuspended in 2 mL of PBS. This RBC solution was diluted in a ratio of 1:50 in PBS and incubated for 15 min at 37 °C. CBS dendrimer solutions were prepared in the range of 2.5–10.240 ppm. In sterile Eppendorf tubes, 20 μL of each solution and 180 μL of incubated RBC solution were mixed, obtaining solutions ten times less concentrated (0.25–1024 ppm). Additionally, controls were included in all of the experiments—Control of all concentrations of compounds (*H*_c_): 20 μL of dendrimers mixed with 180 μL of PBS 1X; negative control (*H*_0_): 20 μL of PBS 1X mixed with 180 μL of incubated RBC solution; and positive control (*H*_100_): 20 μL of TRITON X-100 at 20% solution mixed with 180 μL of incubated RBC solution. All samples were incubated for 2 h at 37 °C. Afterward, the tubes were centrifuged for 15 min at 800 g, and free hemoglobin content (supernatant) was isolated into 96-well microplates. Hemolysis was evaluated by measuring the absorbance at 540 nm. The percentage of hemolysis was calculated from the formula:

$$H(\%)=[(H_{x}-H_{c})/(H_{100}-H_{0})]\times 100$$

where *H*_x_ is the absorbance of the sample.

#### 4.4.2 Hemagglutination Assay

Hemagglutination was determined by resuspending the free hemoglobin content of the original Eppendorf tubes containing each treatment and blood in PBS. 200 μL of the suspension was transferred into a new round-bottom 96-well plate and kept at room temperature overnight. The agglutination of blood was observed, where negative results (no hemagglutination) appear as a compact pellet in the center of the round-bottom plate and positive results (hemagglutination) appear as a diffuse pellet across the bottom of the well. Concanavalin A (2.1 mg/mL) was used as the positive control, and PBS solution was used as the negative control. Each assay was performed in triplicate and repeated three times for each CBS dendrimer and concentration. The data were expressed as the median of three replicates.

Selectivity of the dendrimers samples to microorganisms is described by using the selectivity index (SI), which is calculated as follows:

Selectivityindex(SI)=(H50(ppm))/(MIC(ppm))

#### 4.4.3 Cytotoxicity in Fibroblast Cells

The compatibility of the designed compounds with eukaryotic cells was evaluated by means of a colorimetric cell viability assay, using rabbit dermal fibroblasts as the cell source. The test was carried out in triplicate with a total of 12 samples assayed for each study group.

Fibroblasts were harvested by the explant method from skin tissue biopsies of 3 specific pathogen-free male New Zealand White rabbits. Animals belonged to another study in which our group was involved, in agreement with the Committee on the Ethics of Animal Experiments of the University of Alcalá, Madrid, Spain (PROEX 047.7/22).^46^ Cells were cultured under controlled conditions (37 °C, 5% CO2) using 3 mL of Dulbecco’s modified Eagle medium (DMEM) completed with 10% fetal bovine serum (FBS) and 1% pen–strep antibacterial mixture (all from Gibco/Life Technologies Corporation, Carlsbad, CA, USA). Media were renewed every 72 h, and cell monitoring was carried out using a Zeiss Axiovert 40C phase-contrast inverted microscope (Carl Zeiss, Oberkochen, Germany). Confluent cultures at the second and third passages were seeded in 24-well plates at concentrations of 6 × 10^4^ cells per well and incubated overnight under conditions described above. Following incubation, the medium was replaced with fresh DMEM containing each MBC of the different compounds previously obtained, and plates were incubated again for 24 h. Cells cultured in DMEM without treatment were included as a control. Then, the medium was discarded, and 500 μL of phenol red-free, FBS-free DMEM containing 10% alamarBlue viability reagent (Bio-Rad, Hercules, CA, USA) was added. Following a 5 h incubation period, four 100 μL aliquots were collected from each well to read absorbance (570 nm, 600 nm) in an iMark microplate absorbance reader (Bio-Rad). Data were analyzed using an online alamarBlue colorimetric calculator specifically provided by the manufacturer, and the results obtained were expressed as the mean percentage viability for fibroblasts exposed to the different CBS.

### 4.5 Formulation of the Topical Antiseptic Cream

The preparation of a water-in-oil (W/O) type cream base with dendrimers was carried out according to the composition of the formula shown in Table 3. For the placebo cream, the percentage of water was 30%, maintaining the rest of the compounds at the same percentage.

**Table 3:** Composition of the W/O Cream Formulation Containing Cationic CBS Dendrimers **2a, 2b,** and **2e** as Active Ingredients

compounds	content (%)
bee wax	15.0
SPAN 80	8.0
mineral oil	47.0
water	29.0
dendrimer (**2a**, **2b** or **2e**)	1.0

Creams were elaborated using cationic CBS dendrimers (**2a, 2b,** and **2e**) as active ingredients, according to the formulation shown in Table 3. In this way, each dendrimer, at 1% w/w, was diluted and mixed with distilled water. Then, the aqueous phase and oil phase were warmed to 70 °C. When both phases reached the temperature, the aqueous phase was slowly added to the oil phase under constant agitation to form the emulsion. Agitation of the emulsion was maintained until room temperature was reached. A stable, uniform cream containing each dendrimer was obtained.

### 4.6 Characterization of Creams

See the Supporting Information.

### 4.7 Antibacterial Activity of Creams

#### 4.7.1 Antibacterial Activity of Creams with Dendrimers (Cream **2a**, Cream **2b,** and Cream **2e**) and Placebo Cream (without dendrimer) Has Been Evaluated against (CECT 425) and Methicillin-Resistant (*MRSA*; CECT 5190) Strains

Bacterial suspensions were diluted to a concentration of 10^6^ CFU/mL. Subsequently, treatments were carried out in triplicate in a 6-well microplate. After that, in each well, 0.1 mL of the corresponding cream was mixed with 3 mL of MH broth and immediately inoculated with 1 mL of the target bacterial suspension. Positive control (inoculum in medium MH broth, without cream) and negative control (MH broth containing cream) were added in all experiments. Samples were incubated for 24 h at 37 °C. Then, the absorbance was measured at a wavelength of 625 nm after 24 h of treatment in a Ultrospec 3100 Pro spectrophotometer (Amersham Bioscience, UK). The results obtained were expressed as the mean absorbance produced by bacterial growth. Additionally, the stability of the antibacterial activity of cream 2a was evaluated after 6 months, keeping the creams stored for this period protected from light and refrigerated at 4 °C.

#### 4.7.2 Agar-Diffusion Assay

100 μL of and *MRSA* culture (10^6^ CFU/mL) were inoculated on Petri dishes, which contained 20 mL of LB-agar medium, and spread on the surface to obtain the lawn. Then, 100 μL of each cream sample (*n* = 3 wells on a plate per cream), which contains dendrimers at 1% w/w, as active ingredients, was added into the wells onto the agar surface (9 mm in diameter). All experiments included a negative control and placebo cream that did not contain the dendrimer. Each treatment was incubated at 37 °C for 24, 48, and 72 h. After each time, diffusion zones provoked by the creams in the agar were measured, and the results were shown as the mean inhibition zone (cm^2^) of three replicates per study group. The antibacterial activity of creams is related to the area of the zone of inhibition obtained, expressed in cm^2^.

#### 4.7.3 Analysis of Dendrimer Release from Creams by Agar-Diffusion Assay

Release of dendrimers from creams in liquid medium has been evaluated based on their antibacterial activity. For that, treatments were carried out in a 6-well microplate. First, in each well, 0.1 mL of each cream was mixed with 4 mL of sterile saline solution and incubated for 24 h at 37 °C. After this time, 100 μL of and *MRSA* (10^6^ CFU/mL) was inoculated on Petri dishes, which contained 20 mL of LB-agar medium, and spread on the surface to obtain the lawn. Next, 100 μL of the corresponding cream-saline combination previously incubated (*n* = 4 wells in a plate per treatment), which should contain the dendrimers released from the creams, was added to the wells on the agar surface (9 mm diameter). All experiments included a negative control and placebo cream that did not contain the dendrimer. Each treatment was incubated at 37 °C for 24 h. This process was repeated following 48 and 72 h incubation periods of the cream–saline combinations. Finally, diffusion zones into the agar of creams were measured, and the results were reported as the mean inhibition zone (cm^2^). The release of dendrimers from creams is related to the area of the zone of inhibition obtained, expressed in cm^2^.

### 4.8 Irritation Test

The assay performed was the EPI-200-SIT (OECD-439), carried out by GAIKER Technology Centre (48170 Zamudio, Bizkaia, Spain). The test system used in this study is the RhE model (Reconstructed Human Epidermis), provided by MatTek Corporation (Reference EpiDerm EPI-212-SIT) (MatTek Europe, Bratislava, Slovakia). Tissues were exposed to the test items for 60 min. Then, each tissue was thoroughly rinsed and transferred to fresh medium, where they were incubated for another 42 ± 4 h. Afterward, the cell viability of the treated tissues was determined by the MTT assay.

#### 4.8.1 Tissue Conditioning

On the day of receipt, EpiDerm tissues were conditioned by incubation to release transport-stress-related compounds and debris overnight. The inserts containing reconstructed human epithelium were placed onto 900 μL of the assay medium in 6-well plates. One 6-well plate per test and reference items were used. The plates were incubated for 1 h at 37 °C and 5% CO_2_. After this time, the inserts were transferred to new wells containing fresh medium and incubated overnight under the same conditions.

#### 4.8.2 Test Item Exposure

Three tissues were used for each test item, as well as for the positive and negative controls. 30 μL of the test item, the negative control (dPBS), and the positive control (SDS 5%) were applied topically onto each single tissue.

After dosing the last tissue, all the insets were incubated for 35 min at 37 °C, 5% CO_2_. After 35 min, the inserts were removed from the incubator and placed into a laminar hood at room temperature for up to 60 min. At the end of the 60-min test item exposure, the tissues were rinsed 15 times with dPBS and submerged 3 times in dPBS. The inserts were then incubated with 900 μL of assay medium for 24 h ± 1; the medium was changed, and then, the inserts were incubated for an additional 18 ± 2 h.

#### 4.8.3 MTT Test

Cell viability was assessed by incubating the tissues with 300 μL of MTT solution (1 mg/mL) for 3 h at 37 °C, 5% CO_2_. After incubation, the formazan crystals were extracted using isopropanol (MTT-100-EXT) for 120 min and quantified by spectrophotometry at a 570 nm wavelength. For each treated tissue, cell viability was calculated. Cell viability was expressed as the percentage of the mean negative control (NC) tissues (mean ± standard deviation).

#### 4.8.4 Calculations

For each tissue, the OD value was corrected by subtracting the blank values (isopropanol). Then, the relative cell viability was calculated as follows:

Relativeviability(%)=[(ODtestitem)/(meanODnegativecontrol)]×100

## Supplementary Material



## Data Availability

The data sets generated during the current study are available from the corresponding author on reasonable request.

## Notes

NMR spectra of synthesized compounds, antioxidant data, complete hemolysis, hemoagglutination and antibacterial assays, characterization of creams, UV spectra of release assay, and skin irritation test data (PDF)
