# Supplementary material for: Antibacterial Creams Containing Cationic Carbosilane Dendrimers for Wound Treatment
Source: ACS Appl Polym Mater. 2025 Jul 23;7(15):10026–37. doi: 10.1021/acsapm.5c01718 (PMC12340762; doi:10.1021/acsapm.5c01718)
Supplement: Supplementary file 1 [file ap5c01718_si_001.pdf]

For Supporting Information

Antibacterial creams containing cationic carbosilane dendrimers for wound treatment

Rebeca Lozano-García,[1,2,#] Sara Quintana-Sánchez,[1,2,#] Selma Benito-Martínez,[2,3,4] Guillermo Torrado,[5] Víctor Guarnizo-Herrero,[5] Borja Martínez-Alonso,[5] Gemma Pascual,[2,3,4] Bárbara Pérez-Köhler,[2,3,4,*] Javier Sánchez-Nieves[1,2,3,*], F. Javier de la Mata[1,2,3]

[1] University of Alcalá (UAH); Department of Organic and Inorganic Chemistry, Research Institute in Chemistry "Andrés M. del Río" (IQAR), Campus Universitario, 28805 Alcalá de Henares (Madrid) Spain.

[2] Networking Research Centre for Bioengineering, Biomaterials and Nanomedicine (CIBER-BBN), Instituto de Salud Carlos III, 28029 Madrid, Spain.

[3] Institute Ramón y Cajal for Health Research, IRYCIS, 28034 Madrid, Spain.

[4] University of Alcalá (UAH), Department of Medicina y Especialidades Médicas, Campus Universitario, 28805 Alcalá de Henares (Madrid) Spain.

[5] University of Alcalá (UAH); Department of Biomedical Science, Faculty of Pharmacy, Campus Universitario, 28805 Alcalá de Henares (Madrid) Spain.

[#] Both authors contribute equally to this work.

[*] Corresponding authors: javier.sancheznieves@uah.es; barbara.perez@uah.es.

S1. Figures and tables

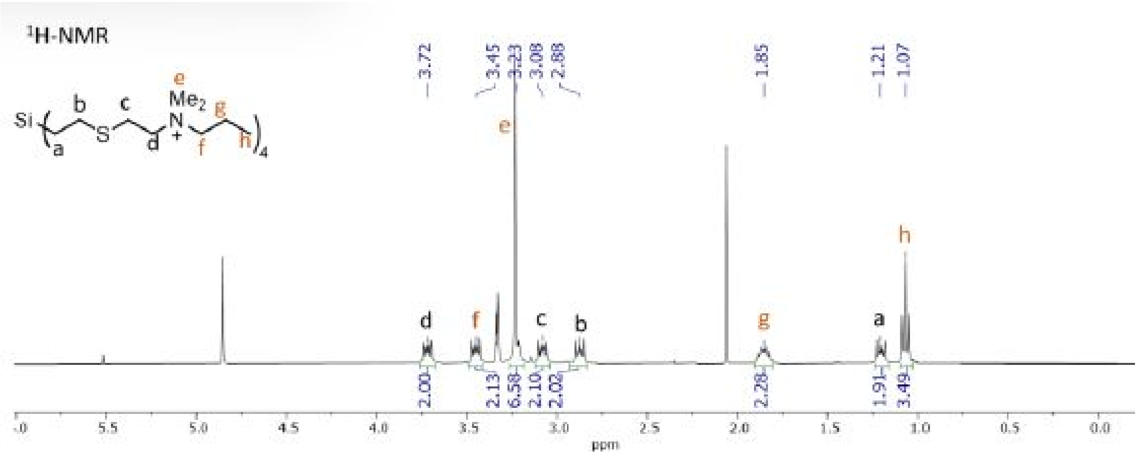

Figure S1. ^{1}H NMR spectrum of cationic CBS dendrimer **1b** (CD_3OD).

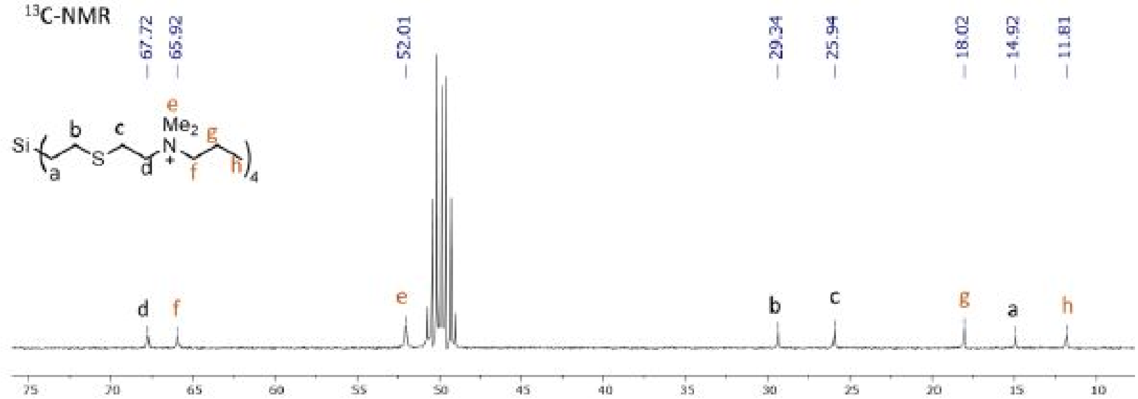

Figure S2. ^{13}C NMR spectrum of cationic CBS dendrimer **1b** (CD_3OD).

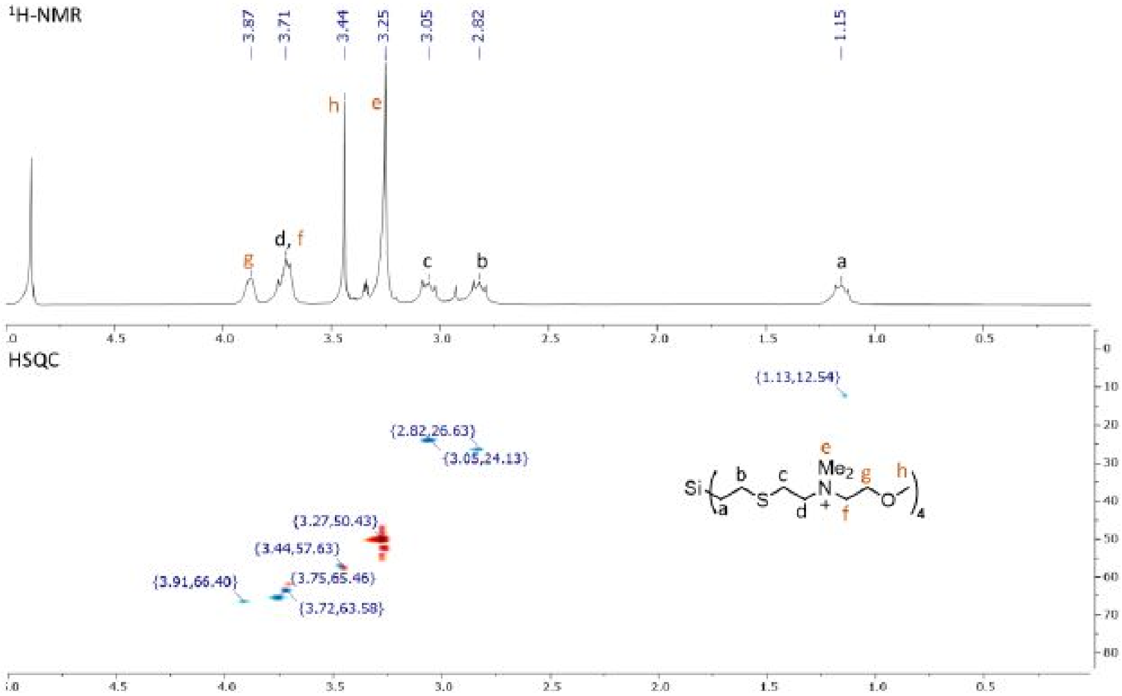

Figure S3. ^{1}H NMR (top) and ^{1}H-^{13}C HSQC (bottom) spectra of cationic CBS dendrimer **1c** (CD_3OD).

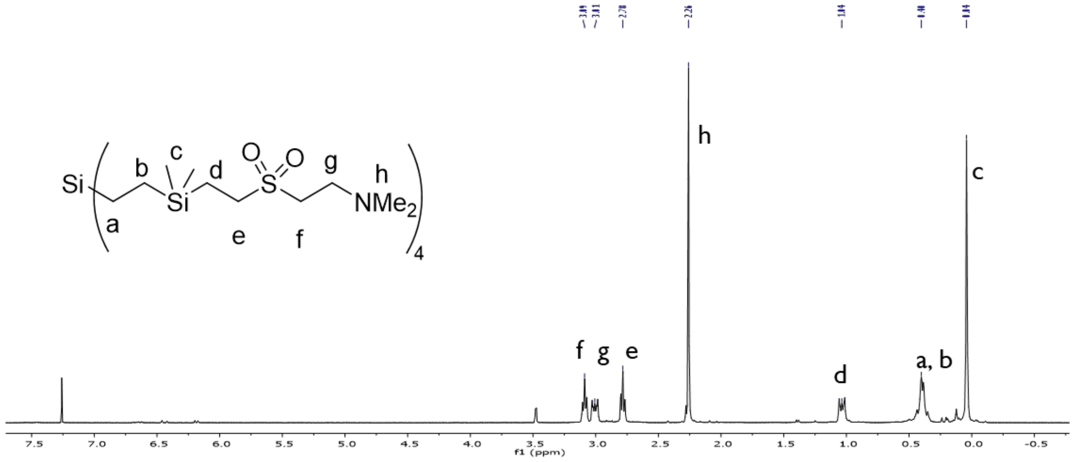

Figure S4. ^{1}H NMR spectrum of neutral CBS sulfone dendrimer **2c** ($CDCl_3$).

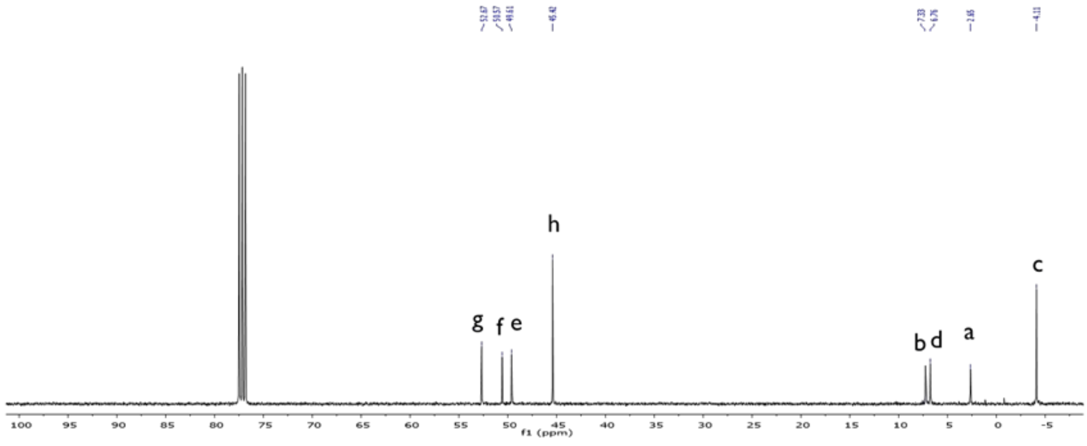

Figure S5. ^{13}C NMR spectrum of neutral CBS sulfone dendrimer **2c** ($CDCl_3$).

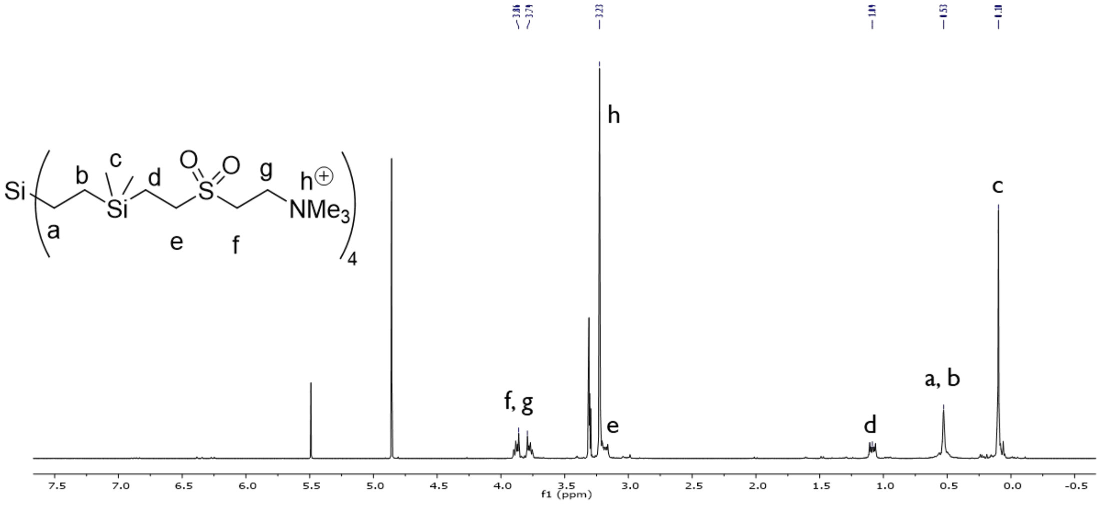

Figure S6. ^{1}H NMR spectrum of cationic CBS sulfone dendrimer **2e** (CD_3OD).

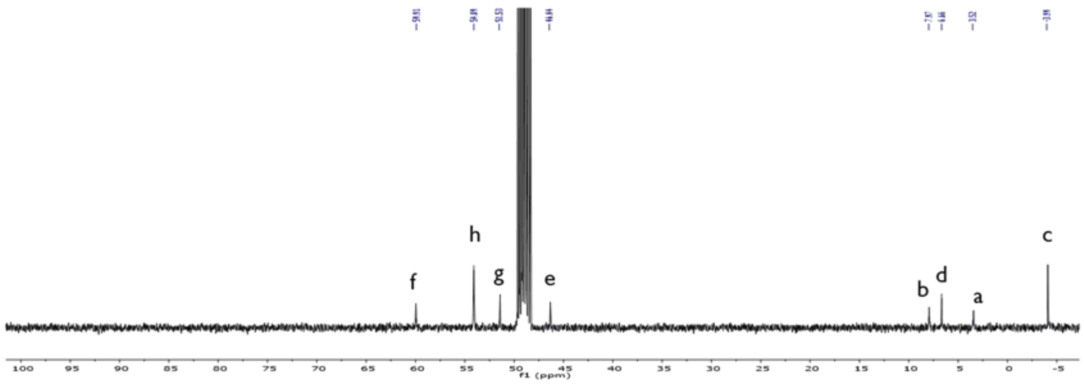

Figure S7. ^{13}C NMR spectrum of cationic CBS sulfone dendrimer **2e** (CD_3OD).

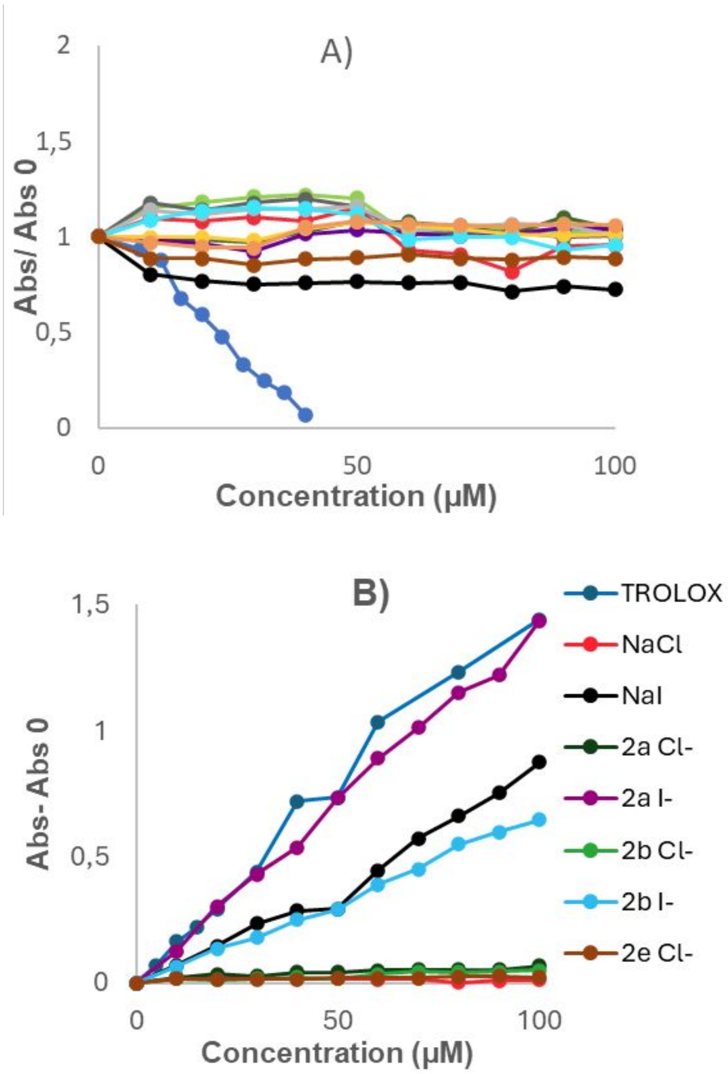

Figure S8. Study of the antioxidant activities in a wide range of concentrations. A) by DPPH assay; Trolox (positive control), NaCl and NaI (anion controls) and cationic CBS dendritic systems **2a**, **b** and **2e**. B) by FRAP assay; Trolox (positive control), NaCl and NaI (anion controls) and cationic CBS dendritic systems **2a**, **b** and **2e**.

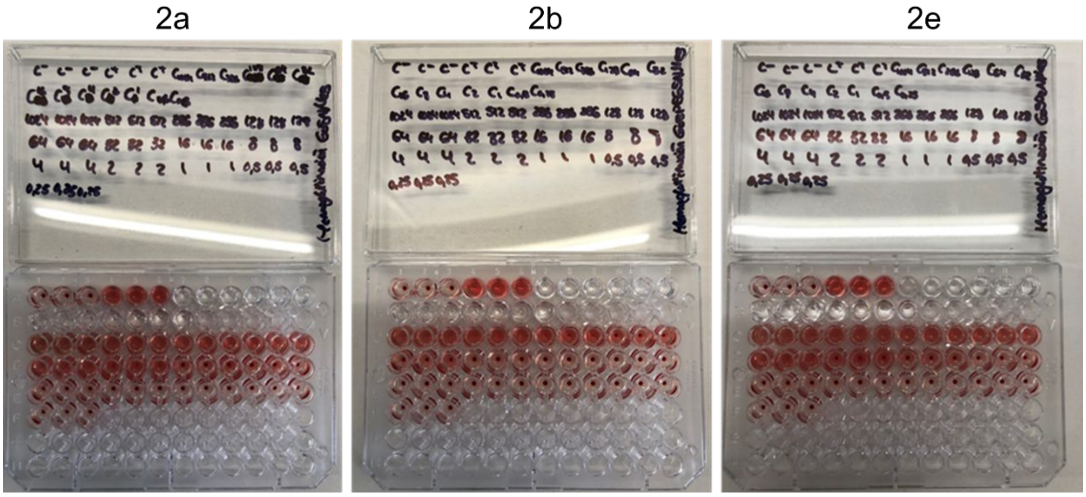

Figure S9. Hemagglutination assay on a microplate of dilutions of dendrimers **2a**, **b** and **2e**. Experiments done by triplicate.

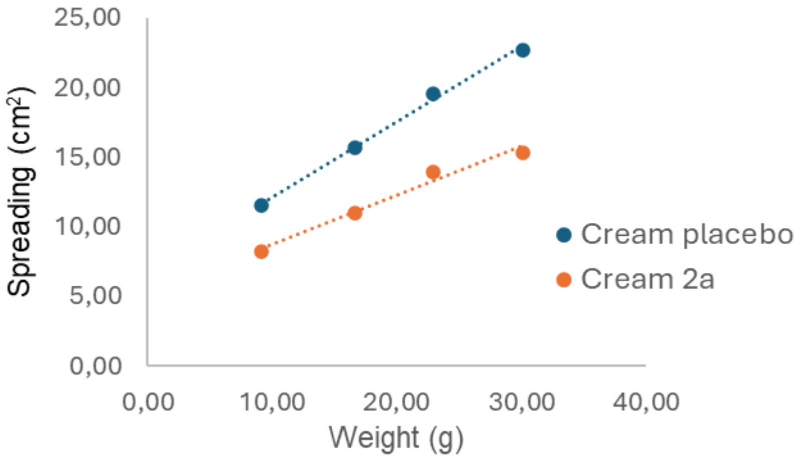

Figure S10. Determination of the spread ability of the placebo and **2a** cream formulations.

A)

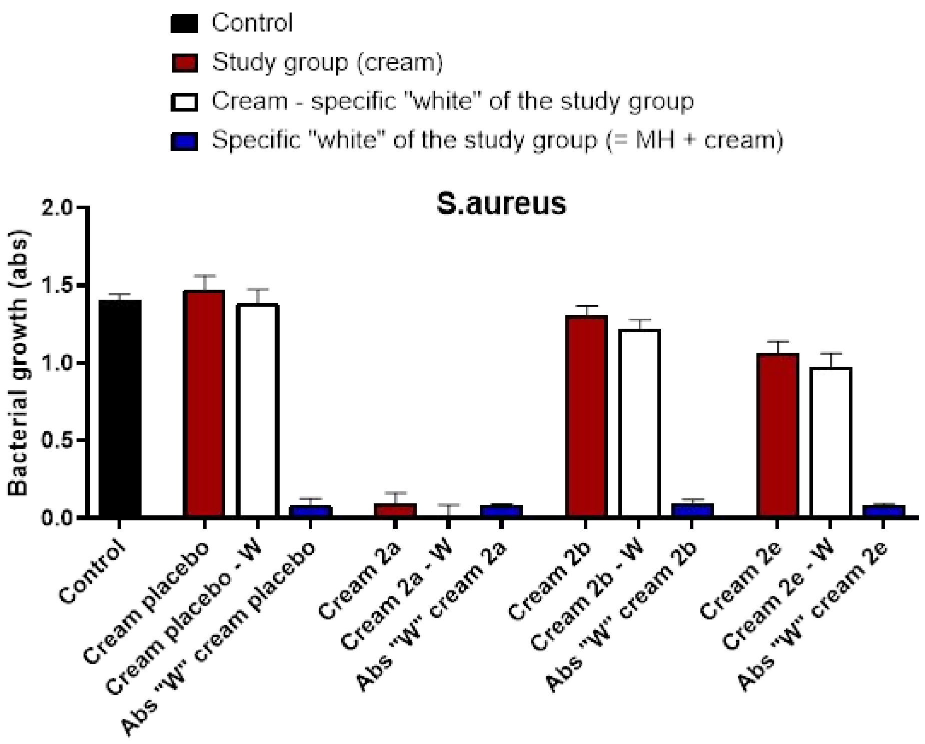

B)

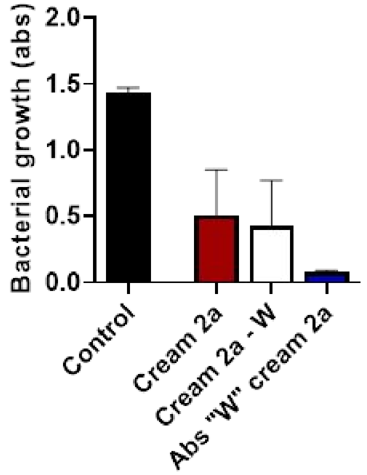

Figure S11. A) Bacterial growth of bacteria (*S. aureus*) in MH medium after incubation with the creams **2a**, **b** and **2e** at 37° C for 24 h. B) Bacterial growth of bacteria (*S. aureus*) in MH medium after incubation with the cream **2a** at 37° C for 24 h after 6 months of cream storage. In black, absorbance of bacteria culture without treatment (positive control); in red, study group (cream), absorbance of bacteria culture after treatment with

cream; in white, absorbance of only bacteria culture after treatment with cream (real activity, red minus blue); in blue, absorbance of cream (negative control).

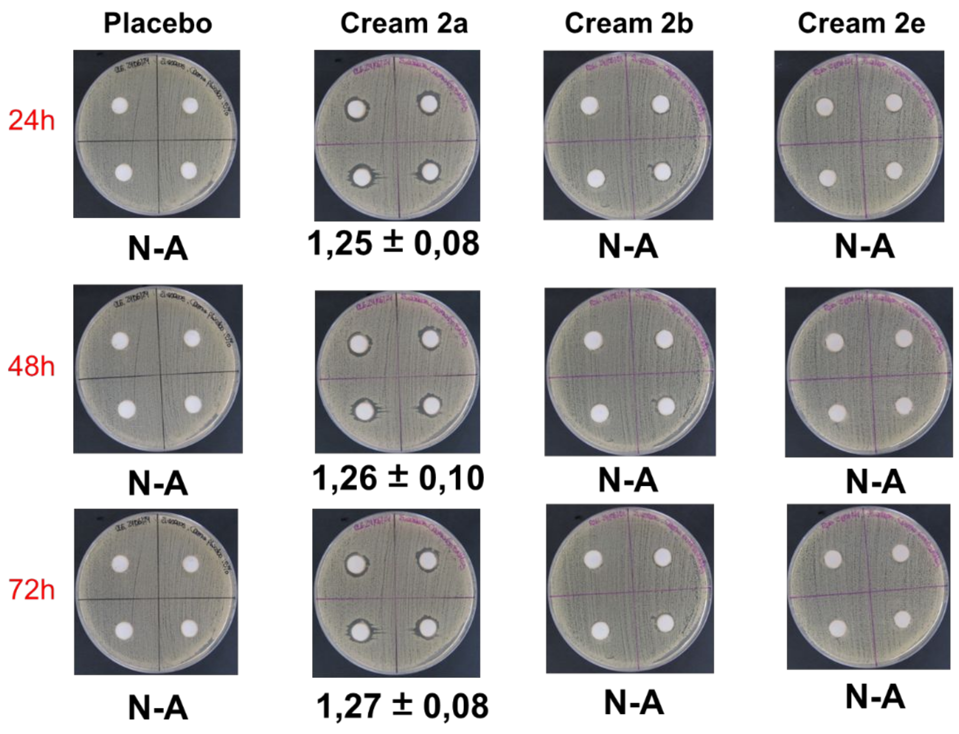

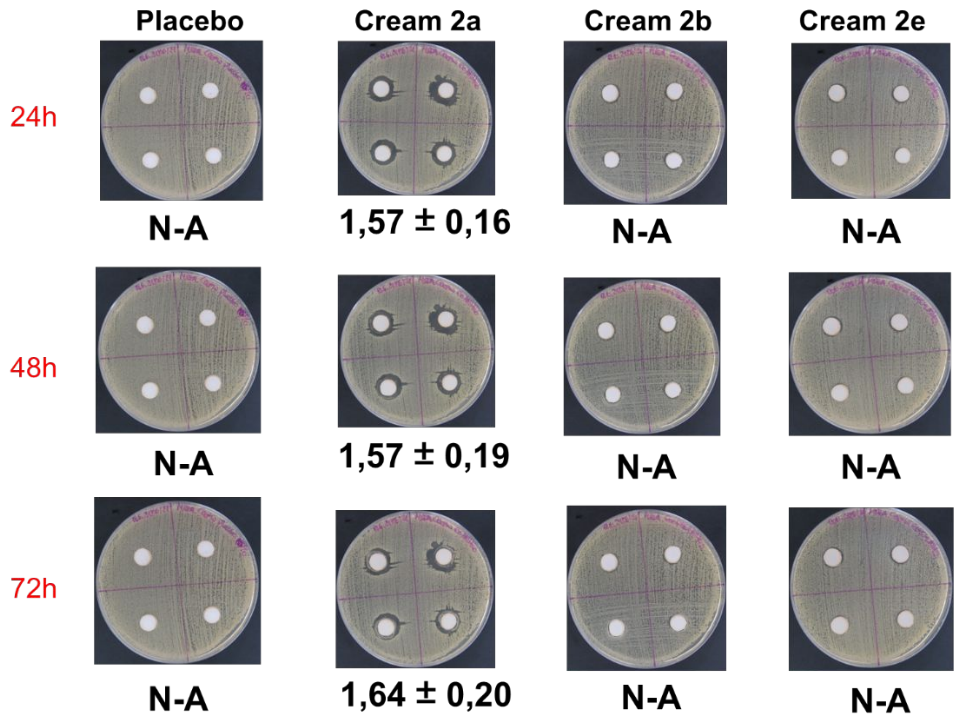

Figure S12. Agar-diffusion assays with topical biocide creams in *S. aureus* (top) and *MRSA* (bottom), where cationic CBS dendrimers **2a**, **2b** and **2e** were added at 1 % w/w. N-A: Non-antibacterial. Area of inhibition in cm^2.

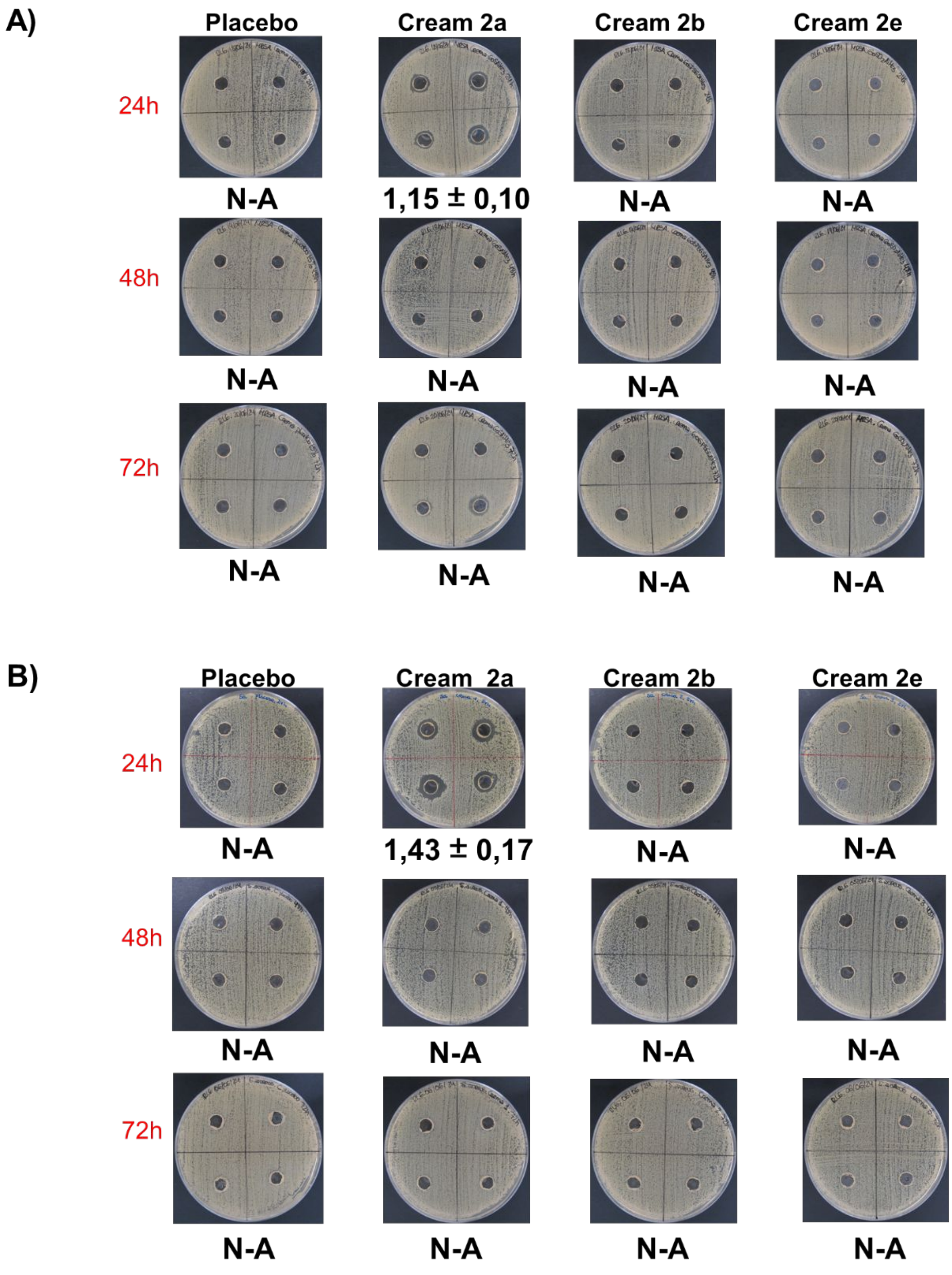

Figure S13. Assay of dendrimer release from creams on agar plates. A) Bacterial growth inhibition of bacteria (*MRSA*) on agar plates after 24, 48 and 72 h incubation. B)

Bacterial growth inhibition of bacteria (*S. aureus*) on agar plates after 24, 48 and 72 h incubation. Non-antibacterial. Area of inhibition in cm^2.

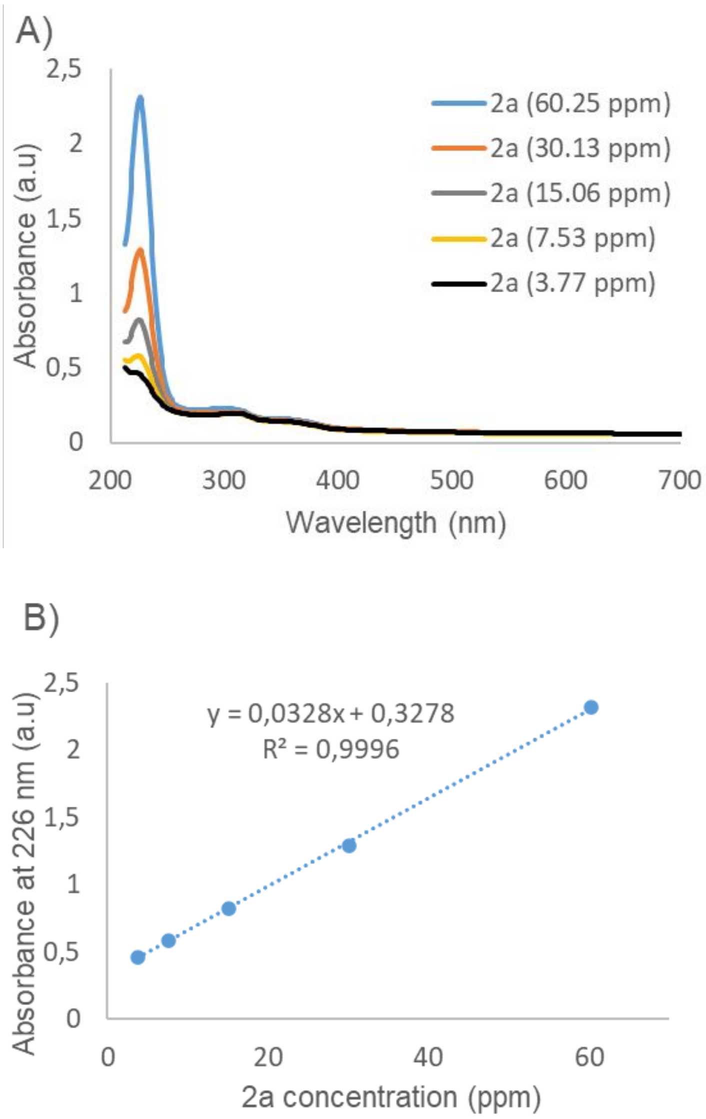

Figure S14. A) UV-vis spectra of different concentrations of dendrimer **2a** using standard **2a** samples in sterile saline medium and B) corresponding calibration line.

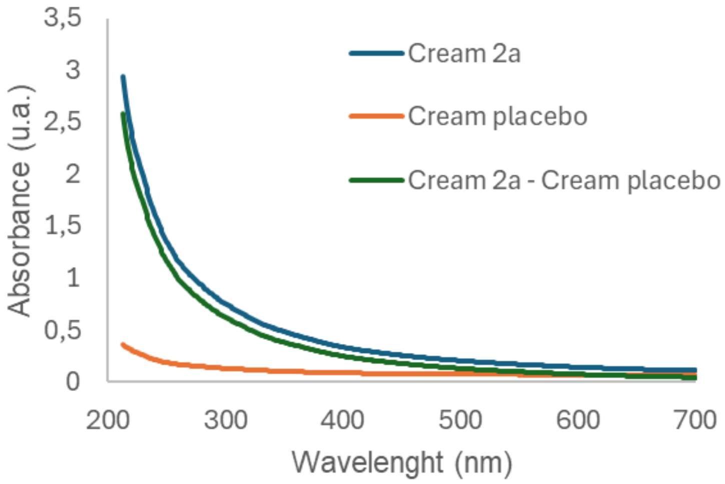

Figure S15. UV-vis spectra of the release assays of dendrimer **2a** from the cream.

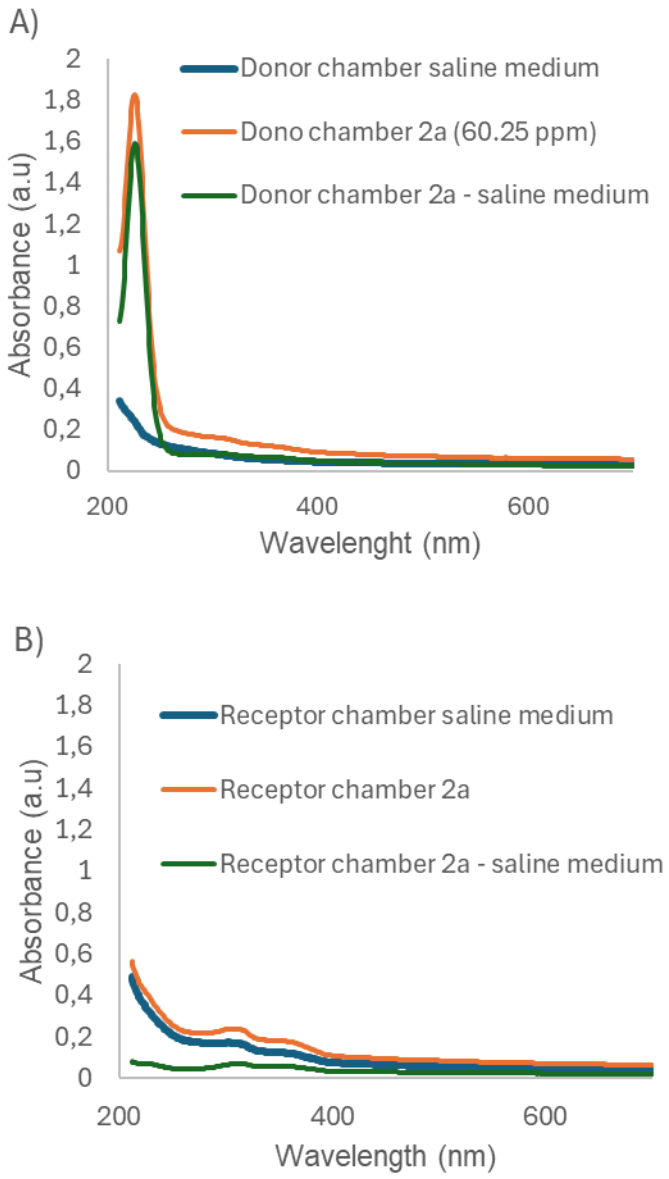

Figure S16. UV-vis spectra of the transdermal passage tests of the dendrimer **2a** in sterile saline medium.

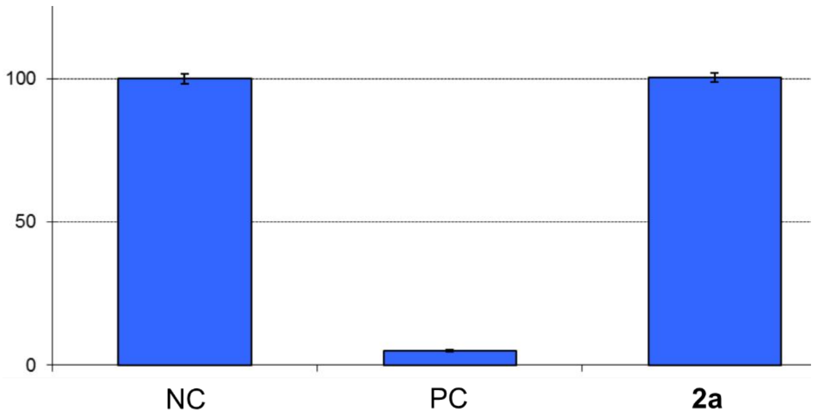

Figure S17. Relative viability (%) of the skin irritation assay for cream **2a**. NC = negative control (dPBS 1X); PC = positive control (5% SDS).

Table S1. Organoleptic properties of placebo and **2a** creams.

**Creams**	**Color**	**Odor**	**Appearance**	**Skin Feel**
**Placebo**	Off-white	Characteristic	Homogeneous without particles	Greasy
**2a**	Beige	Characteristic	Homogeneous without particles	Greasy

Table S2. pH and density of placebo and **2a** creams at 25 ºC.

**Creams**	**pH**	**Density (g/cm^3)**
**Placebo**	5.44	0.91
**2a**	2.10	0.87

Table S3. Determination of spreadability factor (Sf) in placebo and **2a** creams in function of total area (cm^2) and total weight (g).

Creams	Total area (cm^2)	Total weight (g)	Sf (cm^2 g^{-1})
**Placebo**	22.73	30.09	0.75
**2a**	15.36	30.09	0.51

Table S4. Data of MTT asays of skin irritation assay and classification as non-irritant (NI) or irritant (I). According to the EU and GHS classification (R38/Category 2 or no Label), an irritant is predicted if the mean relative tissue viability of three individual tissues exposed to the test item is reduced below 50% of the mean of the negative controls.

	OD	viabilities (%)	CV (%)	Classification
Negative control (NC)	1.890 ± 0.034	100.0 ± 1.78	1.78	NI
Positive control (PC)	0.095 ± 0.004	5.0 ± 0.22	4.33	I
Cream **2a**	1.898 ± 0.029	100.5 ± 1.51	1.50	NI

S2. Characterization of creams

Physical properties. Black formulation (cream placebo without any active ingredients) and drug-loaded formulation (cream 2a) were tested for physical appearance, color, odor and skin feel. These characteristics were evaluated by visual observation. Skin feel was tested by pressing a small quantity of the formulated cream between the thumb and index finger.

Spreadability test. Spread ability of the formulations was determined by measuring the spreading diameter of 0.16 g of sample between two horizontal glass plates (8 cm × 4 cm) after one minute. The weight of top glass plate was 9.16 g. Then applied different weights to the upper plate were 7.44, 13.78 and 20.93 g. The results were

expressed as spread ability of the sample due to the applied weight, according to the equation below:

$$Ei = d^2 \frac{\pi}{4}$$

where: Ei = spread ability of the sample weight for a given i (cm^2); d = diameter (cm).

Spread ability factor (Sf) was calculated using the following equation:

$$Sf = \frac{A}{W}$$

where: Sf = spread ability factor; A = total area (cm2); W = total weight (g).

pH. pH of each cream was determined using a digital pH meter (Mettler-Toledo FiveEasy, Greifensee, Switzerland). It was carried out by measuring only the water phase of each cream at 25 ºC, before combining both phases into an emulsion. This is because in W/O emulsions it is necessary to adjust the pH of the aqueous phase before mixing the aqueous and oily phases.

Density. Density of each cream was obtained by taking 5 mL of each cream with a syringe, avoiding the entry of air, and then weighing the syringe previously tared.

Rheological behavior analysis. The viscoelastic properties of creams were measured using a Discovery Hybrid Rheometer 10 (DHR-10) from TA Instruments (New Castle, DE, USA) using parallel-plate geometry (40 mm diameter) with a 0.9 mm gap at 35 ºC. The samples were placed in appropriate amounts to entirely fill the space between the dishes. The program consisted of an amplitude sweep (0.05 to 500 % strain at 1.6 Hz), a frequency sweep (0.01-50 Hz at 0.1 % strain) and a flow sweep (0.0001 to 1000 s^{-1}). A minimum rest period of 3 min was applied between each section.

S3. Quantification of the dendrimer released from the cream

The quantification of dendrimer **2a** was performed employing a UV-vis spectrophotometer (PerkinElmer Lamba 35). The maximum concentration of dendrimer **2a** present in the cream was dissolved in sterile saline medium and measured from 200-

700 nm to find its maximum wavelength. Then, different concentrations of dendrimer **2a** were prepared to perform the calibration curve.

Next, the treatments were carried out in a 6-well microplate. First, 0.1 mL of each cream (placebo and **2a**) was mixed with 4 mL of sterile saline in each well. The samples were then incubated for 24 h at 37 ºC. After this time, the absorbance of the liquid medium in which each cream was placed was measured in UV-vis. The amount of dendrimer **2a** released was calculated using the calibration curve. The absorbance of the cream itself was also evaluated considering that of the placebo as a blank.

S4. Transdermal Passage

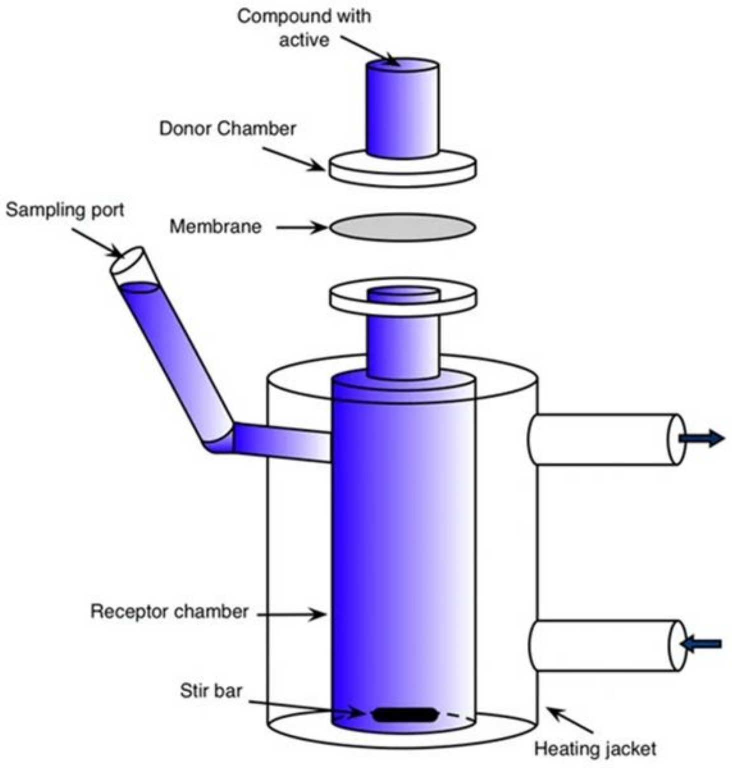

Figure S18. Franz diffusion cell and direction of the flows.

Kinetic tests for in vitro permeation to assess transdermal passage were conducted using Franz cell. Fig. S17 illustrates a Franz cell, consisting of an upper compartment (donor) where the sample under investigation (the active ingredient) was placed. The lower compartment (receptor) contained a liquid medium, which in this instance was a sterile saline solution. A membrane, specifically the 25 mm Strat-M membrane, was positioned between the two compartments.

The Strat-M membrane is designed to replicate the structure of human skin. It consists of two layers: polyether sulfone and polyolefins, along with a blend of synthetic lipids that provide specific resistance to the penetration of substances [1]. Each cell has a contact area of 0.76 cm^2, and the volume of the donor chamber is 3 mL.

The lower compartment has a volume of 12 mL and features a sampling arm for collecting samples to analyze the concentration of the active ingredients that have permeated the membrane. The cell is equipped with an outer jacket and a thermostatic bath to maintain a constant temperature during experiments.

It is essential that during the assay the membrane and the solution in the receptor compartment maintain continuous and full contact, ensuring that the transfer area remains constant.

For the assay, two cells were arranged in series at 37 ºC, which is the generally accepted temperature for skin surface studies, with agitation at 400 rpm for 24 h. In one of them, 3 mL of sterile saline medium was placed on the donor chamber and 12 mL of the same medium on the receptor chamber, to be used as a blank. In the other, 3 mL of dendrimer 2a solution at the maximum concentration set in the cream were placed on the donor chamber and 12 mL of sterile saline medium were placed on the receptor chamber. After this time, the amount of dendrimer 2a remaining in the donor chamber and how much it had managed to cross the membrane was analyzed by UV-vis.

References

[1] A. Haq, M. Dorrani, B. Goodyear, V. Joshi, B. Michniak-Kohn, Membrane properties for permeability testing: Skin versus synthetic membranes, Int. J. Pharm. 539 (2018) 58–64.
